# The ketogenic diet is not for everyone: contraindications, side effects, and drug interactions

**DOI:** 10.1080/07853890.2025.2603016

**Published:** 2026-01-04

**Authors:** Damian Dyńka, Łukasz Rodzeń, Mateusz Rodzeń, Dorota Łojko, Hanna Karakuła-Juchnowicz, Georgia Ede, Żaneta Grzywacz, Katarzyna Antosik, Shebani Sethi, David Unwin

**Affiliations:** ^a^ Institute of Health Sciences, Faculty of Medical and Health Sciences, University of Siedlce, Siedlce, Poland; ^b^ Rodzen Brothers Foundation, Wieleń, Poland; ^c^ Department of Psychiatry, Poznan University of Medical Science, Poznan, Poland; ^d^ Department of Psychiatry, Psychotherapy and Early Intervention, Medical University of Lublin, Poland; ^e^ Independent Researcher, Amesbury, Massachusetts, USA; ^f^ Faculty of Production Engineering and Logistics, Opole University of Technology, Opole, Poland; ^g^ Metabolic Psychiatry, Department of Psychiatry and Behavioral Sciences, Stanford University School of Medicine, Palo Alto, California, USA; ^h^ Faculty of Health Social Care and Medicine, Edge Hill University, Ormskirk, UK

**Keywords:** Ketogenic diet (KD), absolute contraindications, relative contraindications, drug interactions, side effects, diseases

## Abstract

**Background:**

The ketogenic diet (KD), initially developed for the treatment of neurological disorders, has gained increasing attention for its potential role in the management of various metabolic diseases. Alongside its expanding clinical use, concerns have emerged regarding its safety, tolerability, and suitability in specific patient populations. This review summarises key contraindications, clinical situations requiring caution, relevant drug interactions, and commonly reported adverse effects associated with KD.

**Discussion:**

Rare absolute contraindications include selected inborn errors of metabolism affecting pyruvate carboxylase activity, carnitine transport or utilisation, fatty acid oxidation pathways, as well as porphyria. Relative contraindications encompass acute pancreatitis, advanced hepatic or renal disease, familial hypercholesterolaemia, and other conditions that may be aggravated by KD-induced metabolic changes, including concomitant use of propofol. Particular caution is warranted in patients with type 1 or type 2 diabetes receiving specific glucose-lowering therapies, pharmacologically treated hypertension, gallbladder disease or prior cholecystectomy, electrolyte disturbances, cardiac arrhythmias, pregnancy or lactation, underweight status, intense physical activity, significant psychosocial stress, or postoperative recovery.

Clinically relevant interactions with medications are reviewed, including sodium–glucose cotransporter 2 (SGLT2) inhibitors, metformin, glucagon-like peptide-1 (GLP-1) receptor agonists, insulin and sulphonylurea derivatives, antiepileptic drugs, diuretics, lipophilic drugs, and corticosteroids. The most frequently reported adverse effects range from transient “keto flu” symptoms (fatigue, headache, nausea) to gastrointestinal disturbances, polyuria, and hypoglycaemia.

**Conclusions:**

KD demonstrates therapeutic potential in the management of a broad range of metabolic and neurological diseases; however, it is not an intervention suitable for all clinical situations. Awareness of existing contraindications, conditions requiring particular caution, and potential drug interactions enables a more responsible, individualised, and safe approach to patient selection and clinical management. In this context, the present paper provides a concise yet comprehensive synthesis to support clinicians and researchers in the rational and effective application of the ketogenic diet in both clinical practice and scientific research.

## Graphical abstract

**Figure:**
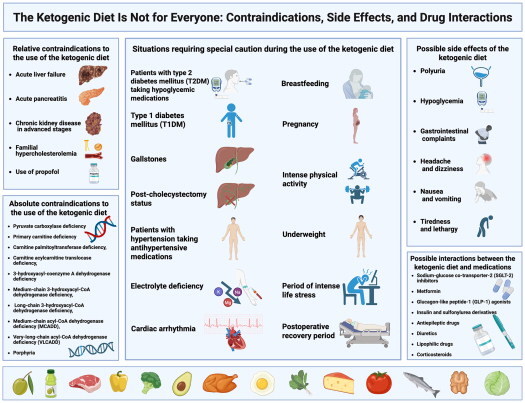

Created in BioRender https://BioRender.com/j0q0zo1

## 1. Introduction

The ketogenic diet (KD) has been in clinical use for over a century since 1921, when it was first used in the treatment of epilepsy [1,2]. However, recent years have seen a rapidly growing interest in this dietary model. A manifestation of this trend is the sharp increase in the number of scientific publications on KD, reflecting the intense search for new potential clinical applications. The beneficial antiepileptic effects of KD raise legitimate questions about the effect of this diet in other brain disorders and diseases, such as Alzheimer’s disease (AD) [3], Parkinson’s disease (PD) [4], multiple sclerosis (MS) [5], migraine [6], and brain tumour [7,8]. There is also a growing number of promising findings on the action of KD in mental illnesses including schizophrenia and bipolar affective disorder [9,10], depression [11], and others [12–16]. For many years, research has also been moving beyond neurology, demonstrating the benefits of KD in other conditions such as type 2 diabetes, where it can often lead to a reduction or complete discontinuation of medication, accompanied by a remission [17–20]. Other areas of research include obesity [21–23], metabolic dysfunction-associated fatty liver disease (MAFLD) [24], cardiovascular diseases [25,26], cancer [27,28], and polycystic ovary syndrome (PCOS) [29,30]. Findings in inflammatory bowel disease (IBD) are also promising, albeit still preliminary [31]. The ketogenic diet is also gaining popularity in the context of sports and physical activity. Although its effectiveness compared to traditional dietary models remains a subject of debate, an increasing number of studies are investigating its impact on physical performance, metabolic adaptation, and recovery [32–34].

The multifaceted (and often successful) clinical application of the ketogenic diet and the growing popularity of this nutritional model calls for a discussion of potential contraindications, side effects and situations in which this particular diet should be used with particular care. Like all other dietary models, the ketogenic diet is not right for everyone. By discussing these concerns, specialists can develop a more responsible and reliable approach to the ketogenic diet.

## 2. Methodology

This manuscript is a narrative review, intentionally chosen due to the heterogeneity of the included evidence (clinical guidelines, mechanistic studies, narrative reviews, case reports, observational studies and expert recommendations), which cannot be synthesised using systematic methods. Searches were primarily conducted in PubMed and Google Scholar. The initial search strategy employed broad combinations of keywords related to the ketogenic diet (e.g. ‘ketogenic diet’, ‘ketosis’), safety considerations (‘absolute contraindications’, ‘relative contraindications ‘, ‘drug interactions’, ‘side effects’, ‘adverse effects’), and clinical conditions potentially affecting ketogenic therapy. To develop a comprehensive review, when individual articles identified a specific contraindication, comorbidity, or safety concern (e.g. acute pancreatitis, primary carnitine deficiency, gallbladder disease, use of SGLT-2 inhibitors), additional targeted searches were performed using the name of the condition or drug together with terms such as ‘ketogenic diet’ or ‘ketosis’. This approach allowed the inclusion of evidence that might not have been captured through broader search strategies.

Selection was based on article titles, abstracts, and full texts.

## 3. The ketogenic diet and the state of ketosis

Due to its complex and often context-dependent nature, the ketogenic diet has been defined in many different ways. The definition describing KD as a dietary regimen leading to increased endogenous production of ketone bodies, resulting in a metabolic state of ketosis is considered the most accurate [35], as it addresses all relevant contexts and nuances. There are a few ways to induce ketosis, one of which is fasting. In a certain way, KD mimics fasting but without the negative effects of starvation. Thus, unlike fasting, it is feasible for long-term use [36–38]. It is important to note that nutritional ketosis typically occurs in the context of low insulin levels, which lead to increased circulating free fatty acids (FFAs), enhanced mitochondrial uptake of FFAs, and elevated ketone body production. This explains why a ketogenic diet must be very low in carbohydrates [39]. Ketosis involves increased oxidation of fatty acids and the resulting ketone bodies (β-hydroxybutyrate, acetoacetate and acetone) are used as the main energy substrate [37]. This makes the KD different from other diets, in which the body derives energy mainly from glucose. The state of ketosis achieved by the diet can be referred to as nutritional ketosis, which in itself shows a therapeutic potential. Some of its benefits are described in Chapter 1.

Nutritional ketosis is a state achieved by following a low-carbohydrate, high-fat and moderate-protein diet. The macronutrient distribution varies depending on the type of KD and the purpose of the diet. A low-carbohydrate, high-fat and normal-protein diet is the most common type. Depending on the patient’s needs and the intended purpose, the share of energy coming from fat and protein ranges from 60% to 90% and from 6% to 30%, respectively [40]. Conversely, according to the 2024 expert consensus [41], the share of energy from carbohydrates is less than 10%, which in practice corresponds to 20–50 g of carbohydrates per day - this is because higher carbohydrate intake requires higher insulin production, which inhibits ketogenesis, as previously mentioned. Protein sources typically used in the ketogenic diet include meat, fish, eggs, offal and seafood. Fat sources include olive oil, avocado, fatty fish, fatty meats, nuts, seeds, MCT oil (typically used as a supplement), butter, lard, or egg yolks, while carbohydrate sources include mainly vegetables and nuts [42]. However, nutritional ketosis is possible in a variety of dietary models, as long as the right proportions of macronutrients are ensured. Those models include a plant-free ketogenic diet, as well as a plant-based ketogenic diet, although it is helpful to include animal products. Another commonly used diet is the Mediterranean version of KD [43]. The term ‘ketogenic diet’ does not refer to a single standardized dietary pattern; rather, its health effects depend heavily on proper formulation. While certain ultra-processed foods such as diet soda and pepperoni may technically comply with ketogenic macronutrient ratios, they are not necessarily conducive to long-term health.

It is essential to clearly distinguish between nutritional ketosis and ketoacidosis, as the two states may be mistakenly conflated. Nutritional ketosis is a physiological and desirable metabolic condition, whereas ketoacidosis represents a pathological state. The most well-known form is diabetic ketoacidosis (DKA), characterized by markedly elevated blood glucose levels (typically >250 mg/dL) and a high concentration of ketone bodies (15–25 mmol/L), levels that are virtually unattainable in nutritional ketosis. In contrast, nutritional ketosis is defined by ketone levels >0.5 mmol/L—typically within a range of a few mmol/L depending on the depth of ketosis—while blood glucose levels generally remain within the normal laboratory range [19,44–46].

## 4. Absolute contraindications to the ketogenic diet (all very rare but important)

Absolute contraindications to the use of the ketogenic diet are rare, however, they carry significant clinical importance, as their presence renders the implementation of this dietary intervention unsafe and, in some cases, potentially life-threatening. Most of these conditions are associated with disorders of fat metabolism and are typically diagnosed early in life, most often during infancy.

### 4.1. Pyruvate carboxylase (PC) deficiency

Pyruvate carboxylase (PC) deficiency is a very rare metabolic disorder, occurring at a frequency of 1 in 250,000 cases [47]. Pyruvate carboxylase deficiency is caused by a mutation in the PC gene (11q13.4-q13.5). The gene plays a role in the conversion of pyruvate to oxaloacetate (an intermediate product in the citric acid cycle and gluconeogenesis). Three types of PC deficiency have been distinguished: type A (infantile PC deficiency), type B (referred to as severe neonatal PC deficiency) and the less common type C (referred to as intermittent or mild PC deficiency). All of these types involve metabolic acidosis [48]. Symptoms of type A include intellectual disability, developmental delay, abdominal pain, vomiting, fatigue, and acid-base imbalance with increased concentrations of lactic acid (lactic acidosis) and increased concentrations of ketone bodies (ketoacidosis). Unfortunately, only some children with type A PC deficiency survive to adulthood. Type B symptoms also include intellectual disability, lactic acidosis and ketoacidosis, as well as hyperammonemia. These are often accompanied by hypotonia, liver failure, seizures, and coma. In these cases, children usually do not survive beyond three months. In contrast, the mildest form, type C, involves mild and intermittent lactic acidosis with normal (or slightly delayed) development and standard life expectancy [49–53].

There have been a series of findings suggesting that the ketogenic diet is contraindicated in each type of PC deficiency as it may aggravate symptoms, for instance by exacerbating metabolic acidosis and further increasing ketone bodies [54–58]. Among other things, this is due to the fact that patients with pyruvate dehydrogenase deficiency are in a way dependent on food-derived glucose, as gluconeogenesis itself is impaired in these individuals [59]. Therefore, in these patients fasting is also contraindicated and their diet should be rich in carbohydrates and protein. Meals should be frequent to prevent the body from becoming dependent on gluconeogenesis [55].

### 4.2. Disorders of fatty acid β-oxidation

Beta-oxidation is a key process for obtaining energy from fatty acids [60], involving gradual degradation of acyl-CoA to acetyl-CoA in the mitochondria. Long-chain fatty acids (LCFA) must be transported into the mitochondria by carnitine and the enzymes CPT1, CACT, and CPT2, while short and medium chains permeate directly [61]. The resulting acetyl-CoA fuels the Krebs cycle, and the generated NADH and FADH_2_ enable ATP production in the respiratory chain. Beta-oxidation becomes critical during starvation, intense exercise and low-carbohydrate diets [60,62]. In individuals with disorders of this pathway, turning to fatty acids as the main energy source is challenging or impossible; restricting carbohydrate supply could lead to serious metabolic complications, therefore, the ketogenic diet is contraindicated in these individuals.

#### 4.2.1. Primary carnitine deficiency (PCD)

Primary carnitine deficiency (PCD) is a rare, autosomal recessively inherited congenital disorder (frequency of 1 per 100,000 cases worldwide). It is caused by mutations in the SLC22A5 gene, which provides information necessary for the synthesis of OCTN2, a protein responsible for carnitine transport into the cell. Carnitine is needed to transport fatty acids into the mitochondria (i.e. the energy centres of the cell), which is particularly important in the heart and muscles, which use fatty acids as the main source of energy. Therefore, mutations in the SLC22A5 gene result in carnitine deficiency in cells and the ensuing consequences [63,64]. Five main spectrums of possible clinical manifestations have been reported in the literature. The first one is metabolic decompensation in infancy (3 months to 2 years) with episodes of hypoketotic hypoglycaemia, hepatomegaly, lethargy, feeding difficulties, elevated hepatic aminotransferase levels and hypoammonemia (induced by fasting or illness, among other things). The second type is childhood myopathy involving the heart and skeletal muscles (with an onset at 2 to 4 years of age); the third type is reduced strength associated with pregnancy or exacerbation of cardiac arrhythmia, the fourth type is fatigue in adulthood and the fifth one is not accompanied by any symptoms [65]. Although the global incidence is estimated at 1 per 100,000 births, it varies significantly from country to country. In Japan, for example, it is 1 in 40,000 births [63], while in China it is as high as 1 in 20,000 births, as a large 2024 meta-analysis involving 10 million newborns demonstrated [66]. The highest incidence (1 in 300 births) has been reported in the Faroe Islands, but the authors of the report point to the efficacy of carnitine treatment for PCD, with patients feeling well 10 years after diagnosis [67]. Levocarnitine (usually at a dose of 100–200 mg/kg/day) is a standard treatment regimen. The therapy is effective, as long as it starts early, before irreversible organ damage occurs [65].

The ketogenic diet is not recommended in primary carnitine deficiency, as discussed in several scientific papers [40,54,68,69]. This is due to the high fat content of the KD, and, as described above, because PCD patients are deficient in cellular carnitine, preventing the efficient use of fats as an energy source. Therefore, contraindications in cases of PCD include high-fat diets, as well as prolonged periods of starvation characterised by increased reliance on fatty acid oxidation [63,65]. In addition, carnitine deficiency inhibits the mitochondrial oxidation of fatty acids to ketone bodies [70], which are the main source of energy in individuals following the ketogenic diet [37]. Moreover, people with PCD are at risk of hypoglycaemia (and hypoketonemia), which may be particularly dangerous if the KD is followed. After a KD meal, blood glucose concentrations rarely spike, and in some cases may even go down [71], which, in the context of hypoglycaemia, is a significant risk. At the same time, due to the nature of PCD, the production of an alternative energy source in the form of ketone bodies is impaired [70]. It is therefore not surprising that in an outpatient setting, PCD patients often use carbohydrate supplementation (mainly in liquid form, orally or by tube). In certain cases, glucose is administered intravenously [65].

#### 4.2.2. Carnitine palmitoyltransferase deficiency

Carnitine palmitoyltransferase deficiency is a rare metabolic disorder caused by a deficiency of either CPT1 or CPT2—enzymes involved in cellular fatty acids uptake and ultimately energy generation [72].

CPT1 (CPT1A) deficiency is an extremely rare, autosomal recessively inherited disorder, with only some 60 cases worldwide reported so far. It is caused by a mutation in the CPT1A gene which encodes carnitine palmitoyltransferase 1 A (CPT1A). The CPT1A enzyme is essential for fatty acid oxidation, as it binds fatty acids to carnitine, which transports them to the mitochondria where they are used for energy generation. Impaired energy production can result in hypoketotic hypoglycaemia (much like in primary carnitine deficiency). Other symptoms of CPT1 deficiency include hyperammonemia, increased blood carnitine levels, increased susceptibility to bleeding, liver failure, seizures, damage to the nervous system, heart and brain, coma and even sudden death [73,74].

Carnitine palmitoyltransferase II (CPT2) deficiency is the most common enzyme disorder, with prevalence estimated at 1 to 9 in 100,000 people [75]). It affects long-chain fatty acid oxidation and is caused by a mutation in the CPT2 gene. It is also the most common cause of recurrent rhabdomyolysis in adults. There are three main phenotypes of primary carnitine deficiency (PCD): the fatal neonatal form, the severe infantile hepatocardiomuscular form and the most common (and mildest) myopathic form, characterised by exercise-induced muscle pain, weakness and myoglobinuria. Interestingly, some individuals may be asymptomatic for most of their lives [72,76]. Much like in CPT1 deficiency, patients with CPT2 deficiency are unable to convert some of the consumed fats into energy [77]. Therefore, the ketogenic diet is considered contraindicated in both CPT1 and CPT2 deficiency [40,78]. The underlying reason is the same as in primary carnitine deficiency. While in PCD the amount of carnitine is insufficient (which impairs energy production from fatty acids), CPT1 and CPT2 deficiency affects the binding of fatty acids to carnitine and their use by the mitochondria as an energy source, as described above. Therefore, a carbohydrate-rich and fat-poor diet is recommended in such patients [72,79]. Frequent meals are also recommended, especially during hypoglycaemic periods, and in cases of severe hypoglycaemia, meals should be accompanied by intravenous administration of dextrose [73]. These strategies prevent the use of fats as an energy source (which is welcome since their oxidation process is impaired), and also prevent hypoglycaemia, to which these patients are particularly vulnerable.

#### 4.2.3. Carnitine-acylcarnitine translocase (CACT) deficiency

Carnitine-acylcarnitine translocase (CACT) is an enzyme found on the inner mitochondrial membrane. It is responsible for carnitine/acylcarnitine translocation across the membrane. Therefore, it is an essential component in the carnitine cycle that regulates the transport of long-chain fatty acids into the mitochondria, where they are beta-oxidised [80,81]. CACT deficiency is an autosomal recessively inherited disorder affecting the beta-oxidation of fatty acids. It is caused by mutations in the SLC25A20 gene [80]. The estimated prevalence in the combined populations of Australia, Germany and the United States is approximately 1 in 750,000 to 1 in 2,000,000 individuals. In Hong Kong and in Taiwan it is estimated at 1 in 60,000 and 1 in 400,000, respectively. Nevertheless, just over 100 cases of CACT deficiency have been diagnosed to date, among which two phenotypes can be distinguished: a severe neonatal-onset form and a later-onset form [81]. The severe neonatal-onset form is far more common and manifests almost immediately after birth. Symptoms include arrhythmias, feeding difficulties, hypotonia, lethargy, hypoketotic hypoglycaemia, elevated liver enzymes, and rhabdomyolysis or hepatomegaly, among other conditions [82–84]. Unless promptly diagnosed and treated, death in the neonatal period is likely, and even if it is diagnosed, the prognosis is still poor, as only a few individuals survive into early and late childhood, and even fewer into early adulthood [82]. In the second, rarer phenotype (a later-onset form), symptoms develop later—after 1 month of age, or sometimes even after 12 months of age. This condition is similar to the first phenotype, but milder. Nevertheless, the prognosis is not promising either, as few people live to adulthood [81–84].

The ketogenic diet is contraindicated in both of these phenotypes, because CACT deficiency impairs the conversion of long-chain fatty acids into energy, and the KD is a high-fat dietary pattern. Similarly, periods of fasting are contraindicated, which is not surprising since they increase reliance on fatty acids [81]. In individuals with CACT deficiency, fasting increases the risk for acute metabolic decompensation [83], and similar symptoms are likely to occur if the KD is followed. This is because maintaining a low LCFA intake on the ketogenic diet is difficult, and in CACT deficiency this particular type of fatty acids is problematic. Therefore, a high-carbohydrate diet is recommended, with more than 60% energy coming from carbohydrates. Conversely, the share of energy coming from LCFAs should be below 10%. Triheptanoin (a special form of fat supplying 25–35% of energy), or medium-chain fatty acids (MCTs) may be used interchangeably [81], as neither MCTs nor triheptanoin require CACT for transport. Therefore, unlike long-chain fatty acids, they are not contraindicated.

#### 4.2.4. 3-hydroxyacyl-coenzyme A (HADH) dehydrogenase deficiency

The 3-hydroxyacyl-coenzyme A (HADH) dehydrogenase enzyme is encoded by the HADH gene. In the mitochondrial matrix it catalyses the oxidation of simple 3-hydroxyacyl-CoA chains as part of the beta-oxidation pathway [85]. HADH deficiency is a rare inherited disorder that prevents the body from converting certain fats into energy, especially during fasting periods [86]. Two main forms of the disorder have been identified: medium-chain 3-hydroxyacyl-CoA dehydrogenase deficiency and long-chain 3-hydroxyacyl-CoA dehydrogenase deficiency.

##### 4.2.4.1. Medium-chain 3-hydroxyacyl-CoA dehydrogenase deficiency (MHADD)

Medium-chain fatty acid 3-hydroxyacyl-CoA dehydrogenase (MHADD) deficiency is a fairly rare disorder. Since it has only been reported in a small number of people worldwide, its prevalence is unknown [86]. As the name suggests, MHADD involves the deficiency of medium-chain 3-hydroxyacyl-CoA dehydrogenase, an enzyme that processes MCFAs and SCFAs. Without a sufficient supply of 3-hydroxyacyl-CoA dehydrogenase, the body is unable to break down certain fats and convert them into energy [87]. Medium-chain and short-chain fatty acids that are not broken down can accumulate in tissues and damage the liver, heart and muscles, causing serious complications. Typical symptoms of the enzyme’s deficiency include hypoglycaemia and lethargy [86]. In this case, the ketogenic diet is contraindicated for a simple reason: the body of a person suffering from MHADD cannot effectively switch to fats as the main source of energy.

##### 4.2.4.2. Long-chain 3-hydroxyacyl-CoA dehydrogenase deficiency

Long-chain 3-hydroxyacyl-CoA dehydrogenase deficiency (LCHAD) results from an isolated deficiency of long-chain 3-hydroxyacyl-CoA dehydrogenase, an enzyme that forms part of the mitochondrial trifunctional protein complex (TFP) [88]. It is a relatively rare disorder, diagnosed in approximately 1 in 250,000 births worldwide. In Poland, the incidence is higher, at around 1 in 120,000 births, with a particularly high incidence in the northern region of Pomerania, where, depending on the source, it is estimated at 1 in 20,000 or even 1 in 16,900 births. This higher prevalence is due to the increased rate of people carrying the pathogenic variant of the HADHA gene, responsible for the development of the disorder [88–90]. The condition usually manifests at any time from a few days to 12 months after birth. In most cases, the phenotype is severe. Possible symptoms and complications include hypoketotic hypoglycaemia, metabolic acidosis, hypotonia, hepatomegaly, encephalopathy, feeding problems, nausea, vomiting, lethargy, arrhythmias and often cardiomyopathy. Sudden heart attack and sudden infant death are also possible, but less common [88,91,92].

The ketogenic diet, as well as other high-fat and low-carbohydrate diets, are contraindicated in people with LCHAD for a simple reason. Namely, these individuals cannot efficiently break down LCFAs, which are abundant in high-fat diets (including ketogenic) [91]. Fasting, caloric deficit during stress, exposure to extreme environmental conditions, intense exercise or even anaesthetics containing high concentrations of long-chain fatty acids (e.g. propofol) are also contraindicated. Conversely, suggested dietary management includes low-fat diets (with particular restriction of LCFAs), increased meal frequency, as well as MCT or triheptanoin supplementation [88,91].

#### 4.2.5. Medium-chain acyl-CoA dehydrogenase deficiency (MCADD)

Medium-chain acyl-CoA dehydrogenase (MCAD) is an enzyme required for the breakdown of medium-chain fatty acids (MCFAs). MCADD is a rare genetic disorder caused by mutations in the ACADM gene that lead to a deficiency of the MCAD enzyme. As a result, MCFAs are not properly broken down [93–96]. The average worldwide prevalence of MCADD is estimated at approximately 1 in 14,600 births. There are no symptoms immediately after birth; the first clinical manifestations usually develop between 3 and 24 months of age. However, some patients may be asymptomatic for life [97]. Symptoms and complications include hypoketotic hypoglycaemia (especially in response to prolonged fasting), which in its turn may lead to lethargy, convulsions, vomiting, coma and even death. Metabolic decompensation can also result in elevated liver aminotransferases, hyperammonemia or chronic myopathy [98].

The ketogenic diet, if based solely on long-chain fatty acids LCFAs with no medium-chain fatty acids (MCFAs), could theoretically minimise the metabolic problem characteristic of MCAD deficiency. Nevertheless, the literature lists the disorder among those in which the KD is absolutely contraindicated [68,99,100]. The reason for this precaution is a) the lack of data on KD use in MCAD-deficient patients, and b) the fact that these patients respond well to glucose-based metabolism. In addition, limiting MCFA intake is much easier with a low-fat or standard fat diet. According to the literature, the recommended dietary strategy is to follow a standard carbohydrate-based diet with fat supply restricted to a maximum of 30% of the daily energy intake and to avoid periods of fasting. If necessary, meal intervals in infancy can be reduced to every 2–3 h. In addition, night feedings, a snack before bedtime or maize starch supplementation at 2 g per 1 kg body weight are recommended to maintain adequate blood glucose levels during sleep [97,98].

#### 4.2.6. Very-long-chain acyl-CoA dehydrogenase deficiency (VLCADD)

Very-long-chain acyl-CoA dehydrogenase deficiency (VLCADD), like MCAD deficiency, affects the breakdown of fatty acids. However, in this case, the body is unable to handle very long-chain fatty acids (VLCFA). The underlying cause of very-long-chain acyl-CoA dehydrogenase (VLCAD) deficiency are mutations in the ACADVL gene [101]. Two forms of VLCADD have been identified: an early (severe) form, which, if not recognised and diagnosed, can lead to cardiomyopathy and be life-threatening; and a later (milder) form, characterised by recurrent bouts of hypoglycaemia [102]. This disorder is also relatively rare, diagnosed in 1:30,000 to 1:100,000 births [103].

The ketogenic diet is listed as an absolute contraindication, which again is a precaution based on a risk-benefit analysis [68,100]. The recommended dietary approach is a carbohydrate-based diet with minimised LCFA intake, increased MCFAs, triheptanoin supplementation, shorter intervals between meals, and avoidance of fasting [103].

### 4.3. Porphyria

Porphyria is an umbrella term for a group of metabolic disorders that cause a deficiency of enzymes involved in the haem synthesis pathway. Haem is a component of haemoglobin, responsible for oxygen transport in the body. If any of those enzymes is not working properly, porphyrins are not converted to haem and accumulate in the body instead. Their excess is the main problem associated with this condition [104]. Porphyria is relatively rare and mainly affects the skin or the nervous system. In most cases it is hereditary, resulting from mutations in specific genes. Based on the symptoms, porphyrias can be divided into acute (mainly affecting the nervous system) and cutaneous (mainly affecting the skin). Among the former, acute intermittent porphyria is the most common, whereas the most prevalent form of cutaneous porphyria (and at the same time the most common overall) is porphyria cutanea tarda, affecting about 5–10 per 100,000 people; erythropoietic protoporphyria is the most common form in children [105]. Symptoms of acute porphyria are often paroxysmal and continue for days or weeks, often several times over a lifetime. They include pain (abdomen, arms, back, legs), digestive problems (constipation, vomiting, nausea), psychiatric concerns (anxiety, hallucinations, confusion, seizures), muscle weakness, paralysis, respiratory problems, urinary symptoms, skin blistering when exposed to sunlight [105,106]. In cutaneous porphyrias, skin exposure to sunlight can lead to blistering (sometimes with secondary infection), increased skin tenderness (injury proneness and healing disorders), scarring and changes in skin colour [105,107].

Many sources list the ketogenic diet as an absolute contraindication for people with porphyria [68,100,108]. This is because low-carbohydrate diets in general (and KD in particular) are among the factors that may increase the risk and the severity of acute porphyria attacks. Low carbohydrate intake increases the activity of mitochondrial ALA synthase (ALAS1), leading to the excessive production of heme precursors such as ALA and PBG. In the case of enzyme deficiencies further down the pathway (e.g. in acute intermittent porphyria), this results in their accumulation, which promotes the development of neurotoxic symptoms and increases the risk of acute porphyric attacks [109–111]. Other potential aggravating factors include periods of starvation, metabolic stress, and losing weight [105]. Therefore, the recommended strategy is a high-carbohydrate (60–70%) diet, avoidance of fasting, and avoidance of very low-calorie diets [112].

A summary of the absolute contraindications to the use of the ketogenic diet is presented in Table 1.

**Table 1.** Absolute and relative contraindications to the use of the ketogenic diet.

Absolute contraindications			
Type of contraindication	Rationale for the contraindication	Frequency of occurrence	Sources
Pyruvate carboxylase (PC) deficiency	Due to impaired gluconeogenesis, patients are largely dependent on dietary glucose and therefore cannot safely follow a fat-based ketogenic diet, nor prolonged fasting.	1 in 250,000	[47,49,55]
Primary carnitine deficiency (PCD)	Due to cellular carnitine deficiency, patients cannot efficiently utilize fats for energy, making high-fat ketogenic diets and prolonged fasting unsafe; they are also at risk of hypoglycemia and impaired ketone production	1 in 100,000 (Japan 1 in 40,000 and China 1 in 20,000)	[63–65,70,71]
Carnitine palmitoyltransferase deficiency (CPT1)	Impaired fatty acid oxidation due to defective CPT1A prevents effective energy production from fats, leading to hypoketotic hypoglycemia; ketogenic diets are therefore contraindicated.	60 cases reported worldwide	[72–74]
Carnitine palmitoyltransferase deficiency (CPT2)	Defective CPT2 impairs mitochondrial fatty acid utilization, reducing energy production from fats and increasing risk of hypoglycemia; ketogenic diets are therefore contraindicated.	1–9 in 100,000	[72,75,77–79]
Carnitine-acylcarnitine translocase (CACT) deficiency	Defective CACT impairs long-chain fatty acid oxidation, limiting energy production from fats; ketogenic diets and fasting increase risk of metabolic decompensation and are contraindicated.	1 in 750,000 to 1 in 2,000,000 (Hong Kong 1 in 60,000 and Taiwan 1 in 400,000)	[81,83,84]
Medium-chain 3-hydroxyacyl-CoA dehydrogenase deficiency (MHADD)	The ketogenic diet is contraindicated for a simple reason: the body of a person suffering from MHADD cannot effectively switch to fats as the main source of energy.	unknown	[86,87]
Long-chain 3-hydroxyacyl-CoA dehydrogenase deficiency (LCHAD)	The ketogenic and other high-fat, low-carbohydrate diets are contraindicated in individuals with LCHAD, as they cannot efficiently oxidize long-chain fatty acids, which are abundant in such diets	1 in 250,000 (Poland 1 in 120,000)	[88–91]
Medium-chain acyl-CoA dehydrogenase deficiency (MCADD)	Although ketogenic diets based solely on long-chain fatty acids could theoretically minimize the metabolic defect in MCAD deficiency, these diets are contraindicated due to the risk of hypoketotic hypoglycemia, lack of clinical data, and the safety and efficacy of carbohydrate-based diets in these patients	1 in 14,600	[97–100]
Very-long-chain acyl-CoA dehydrogenase deficiency (VLCADD)	In VLCAD deficiency, impaired oxidation of very-long-chain fatty acids prevents effective energy production from fats, increasing risk of hypoglycemia and cardiomyopathy; therefore, ketogenic diets are contraindicated.	1 in 30,000 to 1 in 100,000	[100–103]
Porphyria	Ketogenic and other low-carbohydrate diets increase ALAS1 activity, leading to accumulation of heme precursors and risk of acute porphyria attacks; high-carbohydrate diets and avoidance of fasting are recommended.	<200,000 cases in the US; porphyria cutanea tarda: 5–10 per 100,000	[105,109–111]
**Relative contraindications**			
Acute pancreatitis (AP)	In acute pancreatitis, impaired pancreatic enzyme production limits fat digestion; high-fat ketogenic diets may worsen malabsorption and exacerbate symptoms	20 to 40 cases per 100,000	[114,116,120–122]
Acute liver failure (ALF)	The ketogenic diet is contraindicated in acute liver failure due to massive hepatocyte loss, organ dysfunction, and lack of evidence for safety or efficacy; it may also potentially exacerbate liver injury	<10 cases per 1,000,000 per year in developed countries	[128,133]
Chronic kidney disease (CKD) in advanced stages	In advanced CKD (G3b–G5), impaired renal function reduces the ability to excrete ketones and maintain electrolyte balance, increasing the risk of metabolic disturbances; therefore, ketogenic diets are not recommended in these patients.	10.6% of adults globally (CKD stages 3–5; stage 3a and 3b not separated)	[136,138]
Use of propofol	Propofol is considered a relative contraindication during the ketogenic diet because it may impair fatty acid oxidation, increasing the risk of metabolic complications such as acidosis, rhabdomyolysis, or seizure aggravation, although serious events like propofol infusion syndrome are extremely rare.	74% of all anesthesia/ sedation; 90% of general anesthesia	[58,151,153,154]
Familial hypercholesterolaemia (FH)	The ketogenic diet is relatively contraindicated in familial hypercholesterolemia because its effects on lipid profiles are unpredictable-depending on the diet’s composition it may exacerbate high LDL-C levels - and overall safety in these patients has not been established.	1 in 200 to 1 in 1,000	[156,163,164]

PC: Pyruvate carboxylase; PCD: Primary carnitine deficiency; CPT1 or CPT2: Carnitine palmitoyltransferase deficiency; CACT: Carnitine-acylcarnitine translocase; MHADD: Medium-chain acyl-CoA dehydrogenase deficiency; LCHAD: Long-chain 3-hydroxyacyl-CoA dehydrogenase deficiency; MCADD: Medium-chain acyl-CoA dehydrogenase deficiency; VLCADD: Very-long-chain acyl-CoA dehydrogenase deficiency; AP: Acute pancreatitis; ALF: Acute liver failure; CKD: Chronic kidney disease; FH: Familial hypercholesterolemia; ALAS1: Aminolevulinate Synthase 1; LDL-C: Low-Density Lipoprotein Cholesterol.

## 5. Relative contraindications to the ketogenic diet

### 5.1. Acute pancreatitis (AP)

Pancreatitis may be acute (which is the focus of this chapter) or chronic. Acute pancreatitis (AP) is an inflammatory response of the pancreas to damage resulting from a variety of aetiological factors, leading to premature activation of pancreatic proenzymes, (mainly trypsin), inside lobular cells. (By contrast, chronic pancreatitis usually develops in response to prolonged ethanol abuse.) The most common causes of acute pancreatitis are gallstones (35–40% of cases, with women >65 years of age being particularly susceptible [113]), and alcohol consumption (30% of cases). Less common forms include autoimmune pancreatitis, hypertriglyceridaemia, genetic mutations, endoscopic retrograde cholangiopancreatography (ERCP) injuries, pancreatic duct injury and certain medications [114,115]. One of the three key diagnostic criteria for AP is a serum lipase or amylase concentration exceeding the normal range by threefold or more [116]. The disease is quite common. In the US alone, it is associated with the hospitalisation of 200,000 to 300,000 patients per year [117,118]. The incidence in Poland is estimated at 72.1/100,000 patients per year, which is one of the highest in Europe [119], while the global annual incidence of acute pancreatitis in the general population ranges from 20 to 40 cases per 100,000 people [120].

The KD is contraindicated in AP because the pancreas plays a key role in fat digestion by synthesising enzymes such as lipase, phospholipase and esterase [121,122]. Increased fat supply could lead to increased stress on the pancreas, and, since pancreatic function is impaired in AP, fat absorption may be affected and symptoms may be exacerbated. Therefore, the standard recommendation is to withhold oral food and fluid intake until abdominal pain, nausea, vomiting, loss of appetite and symptoms of intestinal obstruction have resolved. In a milder course, easily digestible, low-fat meals may be recommended [114,116]. Importantly however, while nutritional management will vary depending on the case [123], high-fat diets are generally discouraged. However, once ultrasound and liver enzyme tests indicate that acute pancreatitis has resolved, or in cases of chronic pancreatitis, the KD may be a reasonable option, but clinical studies are needed to confirm this.

### 5.2. Acute liver failure (ALF)

Acute liver failure (ALF) is a severe, rapidly progressive condition that leads to hepatic encephalopathy and synthetic dysfunction within 26 weeks or less, and is accompanied by an INR (International Normalized Ratio) of ≥1.5 in patients without cirrhosis and without previous liver diseases [124]. The most common causes of ALF are viral and drug-induced hepatitis. Less common causes include sepsis, poisonous mushrooms, hypoxia-induced liver injury, acute Budd-Chiari syndrome, autoimmune hepatitis, or heat stroke [125,126]. The pathophysiology of ALF is dependent on the initiating factor, but in most cases there is massive hepatocyte death by necrosis or apoptosis, leading to organ failure [124]. Acute liver failure is a rare clinical syndrome, with an incidence of <10 cases per million per year in developed countries. In the U.S., about 2,000 cases are diagnosed annually. ALF often affects younger individuals and carries high morbidity and mortality. It is more common in developing countries due to higher rates of viral hepatitis. The prognosis can be extremely poor and liver transplantation may be required [127,128].

With some liver diseases, KD may have a beneficial effect on the clinical course. One example is metabolic dysfunction-associated fatty liver disease (MAFLD), where KD is not only safe, but may even be one of the more effective therapeutic options [24,129]. Furthermore, the authors of one study [130] showed that KD is promising even in the dietary treatment of patients with nonalcoholic steatohepatitis (NASH), currently more commonly referred to as metabolic dysfunction-associated steatohepatitis (MASH) [131]. There is also a published case study of 2 patients with end-stage liver disease (ESLD) and obesity who were told to reduce body weight in order to qualify for liver transplantation [132]. The patients were put on a very low calorie ketogenic diet (VLCKD) with an energy value of approximately 800 kcal per day. The VLCKD was shown to be well tolerated and safe, and one patient had such a significant improvement in liver function that he could be removed from the liver transplant waiting list. Importantly however, the diet followed by those two patients was not a classic ketogenic diet. Although it did induce a state of ketosis, it was essentially a reduction diet which is not the same as a typical normocaloric ketogenic diet. Furthermore, ESLD involves a chronic, rather than acute, liver failure. For acute liver failure, however, there is no evidence to demonstrate whether KD is effective or not. Therefore, based on indirect evidence and on the pathophysiology of ALF, we feel it is reasonable and responsible to assume that KD is contraindicated in this liver condition, at least until more well-designed clinical studies on the subject become available [133].

### 5.3. Chronic kidney disease (CKD) in advanced stages

Chronic kidney disease (CKD) is a condition characterised by gradual kidney damage that impairs filtration over long periods of time. As filtration becomes impaired, the body accumulates toxins and unnecessary metabolic products, which can lead to multiple systemic complications [134]. Based on the glomerular filtration rate (GFR), which is measured in ml/min/1.73 m^2^, CKD is divided into six stages: G1 (GFR ≥90), G2 (GFR 60–89), G3a (GFR 45–59), G3b (GFR 30–44), G4 (GFR 15–29) and G5 (GFR <15). In stage G1, filtration may be normal but other abnormalities are present, while G5 indicates end-stage renal failure [135]. Chronic kidney disease (CKD) has a high global prevalence, with an estimated 13.4% of adults affected across all stages. Stages 3–5, which include moderate to advanced disease, affect approximately 10.6% of the adult population globally; however, stage 3 was not subdivided into 3a and 3b, and stages 4 and 5 are relatively rare (0.4% and 0.1%, respectively) [136].

It is worth noting that the first three stages (1 to 3a) represent early and mild renal dysfunction, while the remaining stages are more advanced, where renal function is significantly impaired up to complete loss of filtration capacity [137].

In stages 1–3, a ketogenic diet is not necessarily contraindicated; in fact, available data suggest the KD can have beneficial effects [138–140], even when compared to conventional low-fat diets and the Mediterranean diet [141]. For example, one study in people with mild CKD (or with normal renal function) found that low-carbohydrate diets resulted in a significant improvement in creatinine levels, among other improvements [142]. Additionally, it is known that the two most common causes of CKD are type 2 diabetes (30%-50%) and hypertension (27.2%) [143]. Indeed, the KD is an effective therapeutic option for the treatment of type 2 diabetes [19] (even demonstrating the capacity to achieve remission) [144], and can effectively lower blood pressure as well [19,145]. Furthermore, the KD can halt the progression of diabetic kidney disease (DKD) [146], which is often significantly associated with CKD [147].

It should be noted that the currently available scientific data on the use of KD in advanced stages of CKD (G3b - G5) is still insufficient. One study [148] investigating six obese patients with advanced diabetic nephropathy (estimated GFR <40 ml/min, urinary albumin excretion >30 mg/d) showed that a VLCKD applied for 12 weeks to reduce body weight also improved glomerular filtration markers, diabetes status, and other risk factors associated with kidney disease progression. In addition, the diet improved some other general health and well-being indicators. In another study [149], five haemodialysis patients who required weight reduction prior to planned kidney transplantation were placed on a low-calorie diet (approximately 950 kcal per day). While not a classic KD, it was characterised by a low carbohydrate content and, according to the authors, was effective and safe in this population.

Interestingly, patients in advanced stages of CKD have a reduced ability to excrete ketones in the urine and cope with acid loads. In addition, they also face deteriorating renal function, impaired excretion of sodium, potassium, magnesium or fluids (often excreted in greater amounts during adaptation to KD) and electrolyte imbalance. For all these reasons, KD is not recommended for these patients [69,138]. Despite preliminary evidence suggesting that KD is safe and effective in individual cases, the available data is not representative and remains insufficient. Therefore, guided by the precautionary principle and concern for patient safety, advanced stages of chronic kidney disease should be considered a relative contraindication to the ketogenic diet until more studies become available.

### 5.4. Use of propofol

Propofol is an intravenously administered drug that, depending on the dose, induces an intended loss of consciousness. For this reason, it is used in general anaesthesia and sedation of patients during medical procedures [150]. It is estimated that propofol is used in 74% of all anesthesia or sedation procedures and in 90% of general anesthesia cases [151]. The exact mechanism of action of this drug is not fully understood, but it is assumed that its main effect is modulation of GABA type A receptors in the central nervous system. Propofol’s calming, sedating, and anaesthetic mechanism is due to its prolongation of the action of γ-aminobutyric acid (GABA), the brain’s primary inhibitory neurotransmitter [152].

When using propofol, the ketogenic diet is considered a relative contraindication [58,68]. Interestingly, however, this contraindication is based primarily on the case of a 10-year-old boy with epilepsy who developed fatal propofol infusion syndrome (a rare but often fatal complication of this drug) after starting the KD [153]. The authors point out that substances such as propofol, which impair fatty acid oxidation (and are themselves presented in a fatty acid-rich emulsion), may pose an increased risk when combined with the ketogenic diet [58]. It is worth citing a 2023 study that analysed 65 patients with a history of propofol anaesthesia (a total of 165 anaesthesias, including 123 boluses and 42 infusions between 2012 and 2022), while following the ketogenic diet. In bolus dosing, four treatments resulted in acidosis, one in rhabdomyolysis, and one in aggravated seizures. Infusions caused one case of acidosis and one case of seizure aggravation. Importantly, not a single case of propofol infusion syndrome was observed [154]. Therefore, propofol is best considered a relative rather than an absolute contraindication to KD.

### 5.5. Familial hypercholesterolaemia (FH)

Familial hypercholesterolaemia (FH) is a group of inherited genetic defects leading to increased concentrations of low-density lipoprotein (LDL-C) and, secondarily, total cholesterol (TC), with elevated LDL-C being one of the main diagnostic criteria [155]. The disease is the most common monogenic metabolic disorder globally. Depending on the region and the source, its worldwide prevalence is estimated at 1 in 200 to 1 in 1,000 people [156]. FH is reported to significantly increase the risk of cardiovascular disease (up to 13-fold increase in the risk of coronary heart disease) and shorten life expectancy in both men and women by 10 to 30 years relative to the control population. The main therapeutic goal is to lower LDL-C levels [157,158].

The effect of the ketogenic diet on patients with familial hypercholesterolaemia has not been studied thoroughly. This is not surprising given that the diet’s effect on the lipid profiles of patients without FH is still controversial. This is because LDL-C and TC levels often decrease in individuals on KD who are losing excess body fat, whereas they may rise significantly in some lean individuals following the same diet (this typically happens in people with the so-called ‘lean mass hyper-responder’ (LMHR) phenotype [25,159]. Preliminary data suggest that, in individuals with a LMHR lipid profile, the course of atherosclerosis may be somewhat different [160], although the question definitely requires further research. As Diamond and co-authors (2024) have noted, the notion that LDL-C is inherently atherosclerotic may be incorrect. In their opinion, the question is nuanced and depends on the type of LDL-C. They also note that the FH (familial hypercholesterolemia) consensus ignores the fact that only a subgroup of people with FH (those who develop coagulation disorders, regardless of LDL-C levels) die prematurely from cardiovascular disease [161]. In addition, these authors cite findings indicating that coronary artery calcification score, a numerical measure that assesses the amount of calcium in coronary artery walls based on computed tomography (CT) scanning, is a better predictor of CVD risk and death than LDL-C levels alone. In one study, about half of patients with familial hypercholesterolaemia (FH) had a CAC score of zero, indicating a low risk of developing cardiovascular disease (CVD), despite persistently high LDL-C levels. In contrast, high CAC values and elevated fasting glucose levels (rather than LDL-C levels alone) were significantly associated with heart disease [162]. Certainly, further research is needed to confirm these findings.

The effects of the KD on patients with familial hypercholesterolaemia is uncertain, although some data suggest possible exacerbation of hypercholesterolaemia and uncertain safety levels in these cases [163]. The authors of another publication also suggest that before someone is classified as an FH patient, the effect of the diet as a potential cause of hypercholesterolaemia should be excluded [164]. It is also worth noting that the KD can affect the lipid profile in an individualised way (often by lowering TG but sometimes by raising LDL-C), which may call for adjustment of statin dosage or type (especially in people with FH).

With safety in mind, given the unknown impact of KD on patients with FH and the many potentially conflicting indirect data that may determine that impact, familial hypercholesterolaemia should be considered a relative contraindication to KD until these questions are resolved.

A summary of the relative contraindications to the use of the ketogenic diet is presented in Table 1.

## 6. Situations in which special care should be taken when following the ketogenic diet

This chapter deals with a selection of the most common situations in which special care should be taken when the ketogenic diet is followed. However, the authors wish to emphasise that these are not the only clinical cases of concern, and that the risks and benefits of the KD should be analysed separately for each individual.

### 6.1. Patients with type 2 diabetes mellitus (T2DM) taking hypoglycaemic drugs

Evidence suggests that the ketogenic diet may be an effective therapeutic strategy in patients with type 2 diabetes mellitus (T2DM) and may help achieve remission in selected cases [19,144,165]. Indeed, it is known that thanks to KD patients with T2DM can reduce and stabilise glucose levels (fasting, after meals and throughout the day), lower HbA1c and insulin levels, reduce body weight, and reduce (or even wean off) diabetes medication, often achieving full remission. These benefits have been confirmed by a number of meta-analyses and systematic reviews [166–172]. These benefits have been recognised by the American Diabetes Association (ADA), in the 2025 version of Standards of Care in Diabetes (in fact, the effect of KD was noted in earlier editions too). The Standards are considered one of the most important sources of clinical recommendations for the management of diabetes. The most recent version indicates that low-carbohydrate and very-low-carbohydrate diets lead to reductions in HbA1c levels and support dosage reductions of glucose-lowering medications in T2DM patients [173]. These beneficial effects of the KD have also been recognised by other organisations, including the Australian Government in The State of Diabetes Mellitus in Australia in 2024 [174]; the Scientific Advisory Committee on Nutrition (SACN); and Diabetes UK in Lower Carbohydrate Diets for Adults with Type 2 Diabetes [175] and others, such as Diabetes UK [176] or ‘Diabetes Canada’ [177].

Patients with T2DM who take hypoglycaemic drugs (e.g. insulin, glucagon-like peptide 1 (GLP-1) receptor agonists, sodium-glucose cotransporter 2 (SGLT2) inhibitors and sulphonylureas but NOT metformin or GLP-1 agonists [178] should be particularly cautious when considering the ketogenic diet. However, this is not because the ketogenic diet is contraindicated (in fact, T2DM may be an appropriate indication for the KD), but because the diet is so effective at reducing blood glucose (as described above) that hypoglycaemia may develop if the pharmacotherapy (previously dosed for higher carbohydrate supply) remains unchanged. Therefore, dose adjustments (and often weaning off) of hypoglycaemic drugs by the clinician required to effectively manage T2DM and its remission in the context of the KD [19,177,179–183]. SGLT2 inhibitors also carry a risk for diabetic ketoacidosis when combined with the KD. This is why the American Society of Clinical Endocrinology suggests that patients stop taking these drugs even before starting a ketogenic diet [184].

### 6.2. Type 1 diabetes mellitus (T1DM)

Type 1 diabetes mellitus (T1DM) is pathophysiologically distinct from type 2 diabetes mellitus (T2DM). In T2DM, insulin is produced, but target cells exhibit resistance to its action, whereas in T1DM, the primary issue is insulin deficiency resulting from the autoimmune destruction of pancreatic β-cells [185].

Patients with T1DM (who are therefore also taking insulin) who consider switching to the ketogenic diet should exercise particular caution [19]. According to the scientific consensus of the Society of Metabolic Health Practitioners (SMHP), and supported by the emerging scientific evidence, low-carbohydrate diets show promising results in T1DM. The authors emphasise the need for education, improved access to comprehensive information, and support from the healthcare team for patients diagnosed with type 1 diabetes in the context of low-carbohydrate diets [186]. Since dietary carbohydrate supply is very low on the KD, blood glucose levels do not rise nearly as much after meals, and often do not rise at all [71]. The ketogenic diet therefore naturally reduces the need for insulin, one purpose of which is to lower blood glucose levels. Close monitoring and appropriate adjustment of insulin doses by a specialist are therefore necessary to reduce risk of hypoglycaemia. On the other hand, there are concerns that people with T1DM who follow a KD are at increased risk for diabetic ketoacidosis (DKA) [187], and a small number of case studies have documented that this can occur [45,188]. However, as a general rule, risk for diabetic ketoacidosis appears to be low in most cases [189]. For example, one study reported no DKA episodes in a T1DM patient who had followed KD for 10 years [190]. The authors of another paper highlighted that the results (including stable glycemia, absence of health complications, no hypoglycemic symptoms despite low glucose levels, no deterioration in physical or mental performance, and no risk of diabetic ketoacidosis) could be reassuring for clinicians who consider switching their T1DM patients to the ketogenic lifestyle [191]. Among T1DM patients using KD, episodes of DKA are clearly sporadic (as they are observed in only a small subset of T1DM patients following a ketogenic diet, most likely as a consequence of improper dietary implementation) [19], but nevertheless should be kept in mind.

### 6.3. Hypertensive patients taking antihypertensive drugs

Hypertension is one of the most prevalent health conditions globally, affecting approximately 1.28 billion individuals aged 30–79 years. It is also the leading cause of premature death worldwide [192], with more than half (51.2%) of individuals with hypertension taking antihypertensive medication to lower blood pressure [193].

A number of scientific papers have demonstrated that the KD lowers blood pressure [25,145] even more effectively than the DASH (Dietary Approaches to Stop Hypertension) diet, including a 2023 randomised controlled trial [194]. The situation is therefore similar to patients taking hypoglycaemic drugs: the high efficacy of the KD may necessitate adjusting (lowering) the dosage of antihypertensive drugs or weaning them off altogether, if clinically indicated. Otherwise, a potential risk of hypotension arises [195]. This is reflected in some preliminary studies demonstrating that reducing or discontinuing antihypertensive medication in some patients is not only possible, but necessary [196]. However, it is worth noting that the relationship between the KD and antihypertensive drugs has not yet been thoroughly researched. Nevertheless, it has been observed that when combined with antihypertensive treatment, the ketogenic diet can significantly lower blood pressure [197]. Therefore, caution is required in hypertensive patients taking antihypertensive medication.

### 6.4. Cholelithiasis

Cholelithiasis is a clinical condition in which crystals composed mainly of cholesterol, bilirubin and bile components, form in the gallbladder or the bile ducts, and it is one of the most common causes of gastrointestinal dysfunction worldwide. Although it is often asymptomatic, when symptoms do occur, they can be acute, chronic, or episodic [198]. For example, biliary colic is the result of mobile gallstones migrating towards the cystic duct and blocking the flow of bile. When the cystic duct is blocked for several hours, cholecystitis occurs. When the gallstones reach the common bile duct, biliary obstruction may develop, leading to jaundice, cholangitis and even pancreatitis, among other complications [199]. Risk factors for cholelithiasis include metabolic disorders, which are estimated to account for approximately 75% of cholesterol stone cases in Western countries. These metabolic disorders encompass conditions such as diabetes, insulin resistance, obesity and hypertriglyceridemia. Risk for cholelithiasis significantly increases when gallbladder contraction frequency is reduced, because this promotes bile stasis, which in turn elevates the risk of deposit formation [198]. This mechanism may be both dependent on and independent from the factors listed above.

According to some sources, based on observations of children with epilepsy who followed the ketogenic diet, this dietary pattern is sometimes considered to increase the risk of cholelithiasis [200]. In fact, an accurate determination of the actual risk in this case remains difficult due to the concomitant use of medication, the presence of comorbidities or disorders, and the varied nature of the ketogenic diet. The state of ketosis is merely one aspect of the KD; the quality of the food consumed may also significantly affect the risks and benefits of this dietary approach. Additionally, the mere presence of symptoms of lithiasis (the risk of which is in fact increased during KD) does not prove causality, as discussed below. However, there are logical arguments, based on the pathogenesis of cholelithiasis and confirmed by human studies, that low-fat diets can promote the development of this disease. This stands to reason, as bile stasis is one of the primary factors in its aetiology and it is fatty foods that initiate gallbladder contraction and prevent excessive bile stasis [201]. Studies in people following low-fat and low-calorie diets for weight loss have shown that in these individuals, the risk of developing cholelithiasis is higher, precisely as a result of bile stasis [202]. It is also noted that even on low-calorie diets, high fat intake can prevent gallstone formation, precisely by inducing gallbladder contractions [203]. At the same time, higher carbohydrate intake, as well as higher glycaemic index and high glycaemic load are known to increase the risk of symptomatic cholelithiasis in men. Some authors emphasise that low-fat and high-carbohydrate diets may not be the optimal dietary recommendation for the prevention of this disease [204].

Paradoxically, given the mechanisms described above, people with cholelithiasis should be particularly cautious when switching to KD. While the risk of developing new stones is probably much lower, because the gallbladder is more strongly stimulated to contract and expel bile on a high-fat KD, existing stones are more likely to migrate into the cystic duct and common bile duct. This can lead to biliary colic, and/or a number of other related symptoms, as described above. The risks associated with the presence of gallstones aside, the increased likelihood of their migration may help empty the gallbladder, which may be beneficial in certain clinical situations. Low-fat diets also carry some risk, as by reducing the chances of migration of existing stones and favouring the development of new ones, they make gallbladder removal surgery more likely. Further research is necessary to better understand the potential risks and benefits of these different dietary patterns.

### 6.5. History of cholecystectomy

Cholecystectomy (gallbladder removal surgery) is currently performed by laparoscopy. According to the literature, some of the indications for this procedure include acute and chronic cholecystitis, biliary dyskinesia, acalculous cholecystitis, gallstone pancreatitis, gallbladder polyps, and symptomatic cholelithiasis, a condition described in the previous section [205]. After cholecystectomy, gallbladder functions related to bile accumulation and ejection into the duodenum are taken over by the liver. Whereas gallbladder contractions release bile infrequently but abundantly, the liver secretes bile continuously but in small amounts [206,207], which is simultaneously associated with an increased risk for a number of liver diseases [208].

In people with no gallbladder, the ketogenic diet is possible, although care must be taken. There have been few studies on people with a history of cholecystectomy who used the ketogenic diet. However, one study described the case of a 5-year-old boy who used this diet for epilepsy and was able to continue this dietary approach after cholecystectomy without further complications [209].

As regards the bile secretion mechanisms after cholecystectomy, several key aspects should be considered by people who consider switching to the ketogenic diet. Firstly, given the continuous but less abundant bile secretion by the liver after cholecystectomy [206], it seems logical to introduce fat gradually and to determine the body’s tolerance. A symptom known as fatty diarrhoea (steatorrhea) is often closely associated with dietary fat intake in these individuals [210], which can be used to help determine an individual’s tolerance for dietary fat. It also makes sense to spread daily fat intake over 3–4 smaller meals per day, as slow-flowing bile from the liver may have more difficulty digesting a daily portion of fat in 1 or 2 meals per day. Additionally, increasing the proportion of medium-chain triglycerides (MCTs) in the diet seems like a good idea too, as MCTs do not require bile for digestion [211,212].

### 6.6. Electrolyte deficiency

Electrolytes are essential minerals for normal bodily functions. They include sodium, potassium, magnesium, calcium, chloride, phosphate, and bicarbonates. Excessively low (as well as excessively high) electrolyte levels interfere with normal body functions and can sometimes cause life-threatening complications [213]. Among all electrolyte disorders, sodium deficiency (blood sodium level below 135 mmol/l), known as hyponatraemia, is the most common [214], and is associated with increased morbidity and even increased risk of mortality [215]. Another common electrolyte disorder is hypokalaemia, or potassium deficiency (defined as a blood potassium level below 3.5 mmol/l). Severe hypokalaemia can be life-threatening, as it can cause fatal arrhythmias or respiratory muscle paralysis. However, in most cases hypokalaemia is fairly mild, with symptoms including fatigue, muscle cramps, weakness, palpitations or constipation [216]. Magnesium deficiency (hypomagnesaemia), defined as a blood magnesium level below 1.46 mg/dl, can cause a wide range of both mild and severe clinical symptoms, from weakness and mild muscle tremor to cardiac ischaemia and even death [217].

People considering switching to KD should first ensure that they do not have electrolyte deficiencies, or even electrolyte levels in the lower range of normal, which may also be clinically insufficient. It is known that temporarily, during so-called keto adaptation (i.e. the early phase of a KD regimen), urinary water excretion is increased (due to reduced water retention in the body and, indirectly, reduced insulin concentrations). Consequently, the body loses more electrolytes such as sodium, potassium, and magnesium. These well-known phenomena represent a perfectly normal physiological response of the body to carbohydrate restriction and the transition to a state of ketosis [218].

### 6.7. Cardiac arrhythmias

Cardiac arrhythmias comprise a broad spectrum of heart rhythm disturbances and abnormalities. Based on heart rate, a distinction is made between bradyarrhythmias (heart rate below 60 beats per minute) and tachyarrhythmias (heart rate above 100 beats per minute); and these are both further subcategorized by origin or associated syndromes, among other factors [219]. Arrhythmias can involve a wide range of symptoms, such as palpitations, dizziness, breathlessness, fainting, chest discomfort, fatigue, anxiety, among others. Some arrhythmias may even be asymptomatic and thus go unnoticed [220].

People with cardiac arrhythmias wishing to switch to the ketogenic diet should take precautions, especially in the initial stages when the body typically loses more water and electrolytes (for details, see section 4.6), because deficiency of electrolytes such as potassium [216], sodium [221] or magnesium [222], can lead to or exacerbate arrhythmias. Consequently, in individuals with abnormal heart rhythms, the negative effects of electrolyte disorders may be more severe [223,224]. Therefore, when switching to KD, people with cardiac arrhythmias should ensure an adequate supply of electrolytes, especially sodium, potassium, and magnesium.

### 6.8. Pregnancy

It is known that maternal nutrition during pregnancy affects the development and health of the child after birth and in adulthood. It is also associated with epigenetic changes in the foetus [225–227]. The ketogenic diet, for example in patients with polycystic ovary syndrome (PCOS), increases the chance of pregnancy, as a 2024 study shows [29]. It has also been established that during pregnancy, ketone bodies easily cross the placenta, reaching the same levels in maternal and foetal circulation [228,229]. The number of studies investigating KD in pregnant women is still too low to conclusively determine the diet’s effect on the course of pregnancy. Another difficulty is that studies on animal models have been inconclusive [225], and irrespective of their findings, results from animal studies cannot be directly extrapolated to the human population. However, a few papers studying pregnant women using the KD have been published. A 2021 study described the case of a 19-year-old woman who had become pregnant while treated with the ketogenic diet for glucose transporter type 1 deficiency syndrome (Glut1DS). Importantly, her child also suffered from Glut1DS and so was placed on the ketogenic diet at birth. The authors highlighted that, at the time of publication, the child was 5 years old and thriving, which in this case suggests no negative effect of the ketogenic diet on prenatal or postnatal development [230]. Other authors published a series of 2 case studies of women with epilepsy who followed the ketogenic diet during pregnancy. The first of these was the case of a 27 year-old woman whose pregnancy progressed without any complications: a healthy baby boy was born with normal Apgar scores, and his neurological development was normal. The woman continued the ketogenic diet after birth, and during breastfeeding. The other was the case of a 36 year-old woman who had been treated with epilepsy medication and who used the KD during pregnancy. She gave birth to a healthy boy with bilateral auricular deformities, whose neonatal hearing screening results were normal. (It has been suggested that the auricular deformities may not be linked to the KD.) The authors concluded that non-pharmacological therapies for epilepsy such as the KD and the modified Atkins diet (MAD) may be effective during pregnancy, but that their safety in pregnancy still needs to be determined [231]. It should however also be noted that the risk of congenital heart defects is known to increase by 8 per cent for every 10 mg/dl of glucose above normal in early pregnancy in women without gestational diabetes [232]. Importantly, the KD effectively reduces fasting glucose and glycated haemoglobin [19], and is not associated with postprandial glucose spikes [71].

It is interesting to contemplate the example of the Inuit, whose diet was historically composed primarily of fatty animal foods, and was therefore naturally extremely low in carbohydrate. Inuit women continued their usual diet even during pregnancy and, in fact, the consumption of seal or caribou meat has long been seen as important for pregnant women’s wellbeing [233]. Although there is still not enough data to conclusively determine the effect of KD on pregnancy [225], available information suggests that a well-formulated KD may be safe in pregnant women. However, switching to the KD during pregnancy can be problematic due to the well-known physiological stress of keto-adaptation [218]. Therefore, unless medically indicated, pregnancy may not be a good time to implement this particular dietary approach. Switching to the KD well in advance of pregnancy would be much safer, as this timing would not expose mother or the developing baby to the stress of the keto-adaptation period. Given these important considerations and the limited clinical data available in this area, the KD should only be used in pregnancy with extreme care, adequate precautions, and clinical supervision.

### 6.9. Breastfeeding

The importance of breastfeeding is indisputable as it offers a number of benefits not only for the child (including a lower risk of metabolic diseases such as diabetes, overweight or obesity, and higher scores on IQ tests in the future), but also for the mother (including a lower risk of metabolic diseases, cardiovascular diseases and even many cancers) [234]. It is estimated that increasing breastfeeding rates to near-universal levels could prevent up to 823,000 deaths annually among children under the age of five [235].

The question of whether the ketogenic diet is safe during breastfeeding will require more research to draw firm conclusions. Marshall et al. recommend avoiding low-carbohydrate diets altogether, with particular emphasis on the ketogenic diet [236]. However, interesting insights can be found in a 2024 systematic review which collected data on carbohydrate restriction (mainly different variants of the ketogenic diet and other low-carbohydrate diets) during lactation. In the studies evaluated, although nursing mothers experienced negative symptoms such as vomiting, nausea, abdominal pain, muscle weakness, fatigue and malaise, no risks to their babies were reported [237]. Furthermore, these outcomes should not be extrapolated to the general population, because the analysed individuals (with two exceptions) were using the KD without professional supervision, and mainly for weight loss, therefore calorie deficits may have contributed to the negative experiences. It should be emphasised that restrictive low-calorie diets are absolutely contraindicated during pregnancy and breastfeeding [238]. A survey of women who had followed a KD while breastfeeding found that most of them appreciated this dietary strategy. However, attention was drawn to the risk of lactation ketoacidosis, which was reported in 1 out of 21 women. Still, this condition is probably not caused by KD alone, but also due to other variables, such as a child’s illness leading to more frequent breastfeeding combined with extremely low food intake by the mother. Some women also noted reduced milk supply associated with the diet which seemed to resolve relatively quickly when additional calories and/or carbohydrates were added [239]. Returning to the example of the Inuit, it is known that Inuit women successfully breastfeed their children for centuries despite very limited access to carbohydrates. This historical example strongly suggests that the human body is able to adapt to a low-carbohydrate dietary pattern, even during lactation [233]. However, it should be noted that the diet and lifestyle of indigenous Inuit women cannot be directly extrapolated to most of the world’s female population.

No firm conclusions can be made on the basis of this limited information. However, it is reasonable to state that a well-formulated ketogenic diet may be safe during breastfeeding, as long as extreme caution is taken and the clinical condition of mother and child is regularly monitored.

### 6.10. Underweight

Most individuals (up to 77%) use the ketogenic diet for weight loss, which is also how it is most commonly perceived [240–243]. This is very likely because the diet has been shown to be superior to high-carbohydrate weight loss diets. KD outperforms other diets in many respects, including appetite and hunger suppression, greater initial weight loss, superior anti-inflammatory effects, better regulation of glycaemia and insulinaemia, and psychological benefits [21]. Some of these effects, although desirable in the context of weight loss, can be highly problematic for underweight individuals who need to gain weight. For example, it is known that individuals wishing to gain weight should eat more, but the KD effectively reduces appetite and hunger [244–246], and the initial rapid weight loss (resulting from the loss of glycogen and water) [21] that occurs with the KD is also undesirable in underweight individuals.

Paradoxically, preliminary findings suggest that the KD may hold benefits for people with anorexia nervosa (AN). For example, a retrospective case series of 3 adults with severe, underweight AN that had been refractory to standard therapy documented that after switching to a ‘carnivore’ KD (comprised entirely of animal protein and fat), complete, sustained remission of AN was achieved, with all 3 individuals gaining more than 20 kg of body weight. This dietary intervention was also associated with reduced anxiety symptoms and improved overall psychological wellbeing [247]. There are also 6 prospective cases of women with weight-restored anorexia nervosa whose residual eating-disordered thinking and other psychological symptoms of AN partially improved in response to a standard KD followed by ketamine therapy [248].

A 2024 paper discussed the potential mechanisms underlying the reversal of AN symptoms in response to KD therapy. Among other things, the authors point to the diet’s effects on the central nervous system, which may attenuate the pathological desire to lose weight and mitigate other symptoms characteristic of eating disorders [249]. Norwitz et al. proposed classifying AN as a ‘metabolic-psychiatric’ condition, indicating that it may respond to treatment with metabolic interventions that exhibit neuromodulatory properties [247].

With the above in mind, a distinction must be made between cases of slight underweight in people simply wishing to gain a few pounds, and AN-a life-threatening metabolic-psychiatric disease. Given the diet’s neuromodulatory properties and other metabolic benefits, the KD may prove an effective tool in the management of AN, much like in other conditions studied in the growing field of metabolic psychiatry [9,250–252]. Conversely, for reasons mentioned in the first paragraph, in cases of simple underweight unrelated to AN, the KD may not be the most appropriate or effective weight normalisation strategy.

### 6.11. Intensive physical activity

The ketogenic diet appears to be well suited to lower-intensity physical activity requiring a balanced or moderate energy demand, but there is no consensus regarding its effect on more intense exercise [253]. One paper reports that for endurance athletes, the literature supports the use of the KD as an effective strategy for reducing body weight and fat mass, suggesting potential improvements in performance at submaximal intensities (∼60%) [254]. The authors of another paper suggest that KD is unlikely to be optimal for improving performance in high-intensity endurance events or activities requiring rapid bursts of carbohydrate-derived energy [255]. However, it is worth noting that there is some disagreement regarding the effect of KD on athletic performance. One of the key concerns is the adaptation to this dietary approach [34,256]. It is estimated that a low-carbohydrate diet must be adhered to for a minimum of four weeks in order to achieve the required energy homeostasis and improve exercise performance. This is yet another observation suggesting the importance of the aforementioned adaptation period [257]. While this particular aspect may be decisive, some studies have looked at athletic performance in non-adapted athletes. A 2025 paper examining the impact of low-carbohydrate diets on athlete performance shed a new light on the officially accepted narrative. According to its authors, their study challenges the key role of carbohydrates in athletic performance and the belief that muscle glycogen (or an adequate supply of exogenous carbohydrates) is key and/or indispensable in maintaining long-term exercise performance. They pointed out that a key factor affecting exercise performance is hypoglycaemia, eliminated by consuming just 10 g of carbohydrate per hour (which improved performance by 22% despite low glycogen availability).

At the same time, water and electrolyte losses are known to occur during exercise (especially if intense), both of which are essential for muscle and nervous system function, among other things [213,258,259]. The ketogenic diet increases both electrolyte and water excretion during the adaptation period [218], which can be detrimental to athletes. This should be kept in mind by everyone switching to the KD in a period of physical activity (especially intense physical exercise).

### 6.12. Periods of intense stress

For several reasons, a period of intense stress is not a good time to switch to the ketogenic diet. Firstly, the period of adaptation to KD is itself stressful for the body, if only because of a number of changes that are triggered by switching to a new energy source (from glucose to ketones), including changes in water-electrolyte balance [218]. Indeed, it is known that in the short-term (<3 weeks), the KD can increase levels of cortisol [260], the stress hormone, and increased cortisol levels are accompanied by psychological stress. As a result, the burden on the body may become excessive [261]. Stress is also often associated with impaired urinary function [262] which plays a particularly important role during keto- adaptation [218]. Cortisol also affects the regulation of ketogenesis, the metabolic pathway through which ketone bodies are produced [39]. At the same time, cortisol also stimulates gluconeogenesis - the conversion of non-sugar precursors into glucose [263] - which in turn may interfere with the normal adaptation to the state of ketosis (depending on the type, intensity and duration of stress), which may once again be linked to the inhibition of ketosis by insulin. The literature even suggests that type 2 diabetes may be triggered by psychological and physical stress. Indeed, it is known that stress can increase glucose levels (hyperglycaemia) and insulin requirements, at the same time promoting insulin resistance [264]. The process of ketogenesis and adaptation to the state of ketosis may therefore be inhibited by elevated levels of glucose. Given anti-ketogenic properties of cortisol, periods of intense stress are not the best times to start the ketogenic diet. However, this question needs to be addressed in a more nuanced way; since obesity itself can be a significant stressor [265], weight reduction that accompanies the KD may reduce stress symptoms [266]. It is important to note, however, that these benefits are primarily observed in chronic stress rather than acute, intense stress.

### 6.13. Postoperative recovery period

Post-operative recovery is a period when the body undergoes a process of tissue regeneration and metabolic adaptation, and returns to physiological homeostasis to restore functional capacity and overall health. During this period, the patient should rest, avoid any activity that could interfere with recovery, and maintain proper hydration and nutrition [267]. Surgery itself can be psychologically stressful, but can also disturb metabolic and physiological processes, impinging on the metabolic pathways of nutrient absorption and digestion that ultimately lead to energy production [268]. Switching to the ketogenic diet at this time is not optimal, as the process of keto-adaptation is in itself a stressor for the body. Combined with the psychological and metabolic stress after the surgery, it carries the risk of excessive burden on the body, as described in section 4.12.

This is also true for patients after bariatric surgery and other surgeries that involve the gastrointestinal tract (e.g. oesophageal, pancreatic, small and large bowel surgeries). In these cases, the issues described above (recovery and postoperative stress) are accompanied (in the long term) by the impact of changes in the GI tract structure. While it is known that a very low-calorie ketogenic diet can be safe and effective for weight restoration in patients after bariatric surgery [269,270], the feasibility of the KD in such patients will depend on the type of surgery and their individual tolerance. Therefore, special caution should be exercised in all such cases.

**Figure 1.**
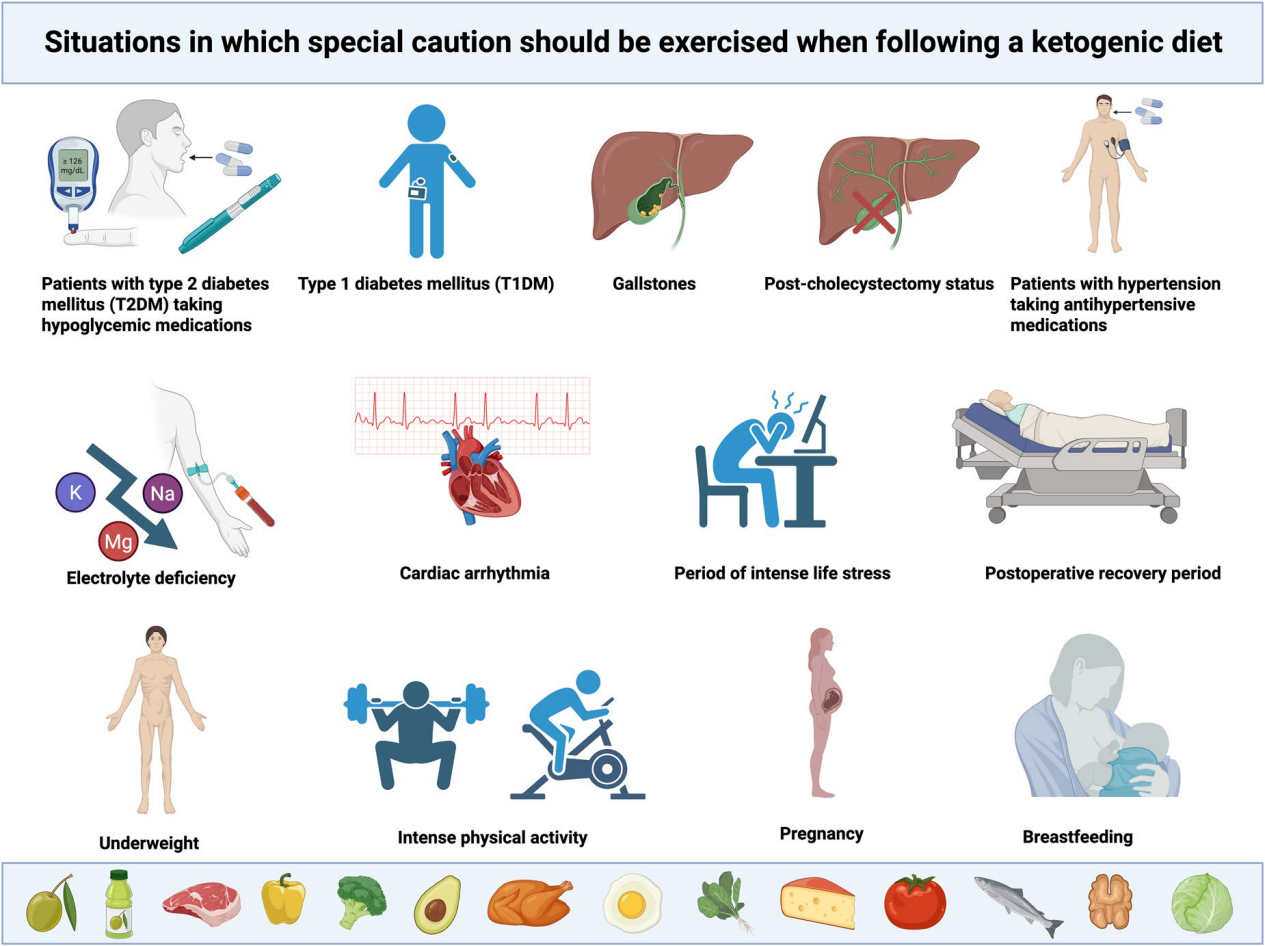
Situations in which special caution should be exercised when following a ketogenic diet Created in BioRender https://BioRender.com/v8tm6qb.

Situations in which particular caution is required when using the ketogenic diet are illustrated graphically in Figure 1

## 7. KD and drug interactions

The ketogenic diet can interact with certain drugs both indirectly - through modulation of common metabolic or physiological pathways - and directly, at the molecular level, affecting drugs’ pharmacokinetics and pharmacodynamics. Some interactions are beneficial, such as enhancing the effect of medications and thus supporting eventual dose reductions. However, adverse interactions are also possible. These may weaken drug efficacy or exacerbate side effects, requiring careful monitoring and individualised therapy. Selected interactions are presented below, but the authors wish to emphasise that, given the multitude of drugs and their mechanisms of action, those described below are certainly not the only possible interactions between KD and medication to be aware of.

### 7.1. Sodium-glucose cotransporter-2 (SGLT-2) inhibitors

Sodium-glucose cotransporter-2 (SGLT-2) inhibitors are a class of drugs commonly used to improve blood glucose control in adults with type 2 diabetes. SGLT2s are proteins involved in the absorption of filtered glucose from the tubular lumen of the kidneys. Thus, inhibitors of these proteins will decrease the absorption of filtered glucose, lower the renal threshold for glucose, and increase urinary glucose excretion [271]. It appears that similar effects can be achieved by restricting carbohydrates in the diet, with a low risk of side effects, as described by the authors of one paper [272]. In contrast, the KD combined with SGLT-2 inhibitors may offer some benefits as well as create some risks.

Firstly, both the KD and SGLT-2 inhibitors promote lower blood glucose levels [19,271], which is beneficial, provided treatment is monitored to avoid hypoglycaemia.

Secondly, both SGLT-2 inhibitors and the initial phase of the KD (keto-adaptation) may disrupt water and electrolyte balance by increasing the loss of water and electrolytes from the body [273]. Importantly, some studies do not clearly confirm the impact of SGLT-2 inhibitors on electrolyte balance, suggesting that the observed effects may be selective and element-specific. For example, one study demonstrated that SGLT-2 inhibitors increased serum magnesium and phosphate levels, with no significant effect on other electrolytes [274]. One report even suggested the risk of hyperkalaemia [271].

Thirdly, some reports point to a possibly greater risk of euglycemic diabetic ketoacidosis (euDKA) when combining KD with SGLT-2 inhibitors [275]. One patient following a carbohydrate-restricted diet developed euDKA after just the first dose of empagliflozin, while another patient who was taking an SGLT2 inhibitor developed euDKA after only 1 week on the ketogenic diet [276].

### 7.2. Metformin

Metformin is a drug used in patients with type 2 diabetes, and in some patients with pre-diabetes. The drug lowers blood glucose concentrations by reducing glucose production in the liver and its absorption in the intestine. In addition, it improves insulin sensitivity. Recently, an increasing number of studies have been published exploringits potential anti-cancer, anti-ageing and neuroprotective properties [277].

The ketogenic diet may act synergistically with metformin, as it also effectively lowers blood glucose concentrations and improves insulin sensitivity [19,277]. The first preclinical study demonstrated a possible beneficial synergy of KD and metformin with respect to anti-cancer effects, specifically in the treatment of triple-negative breast cancer (TNBC). Indeed, it is known that cancer cells require abnormally high concentrations of glucose, so it would be logical to reduce glucose intake and blood glucose levels. The study found that by lowering blood glucose concentration, the integrated actions of KD and metformin significantly inhibited tumour proliferation and improved overall survival [278]. Importantly, the combination of KD and metformin was shown to have a better effect in this context than either of these strategies alone.

### 7.3. Glucagon-like peptide-1 (GLP-1) agonists

GLP-1 (glucagon-like peptide-1) is an endogenous incretin hormone synthesised in the L cells of the small intestine in response to food intake. It has a multifaceted physiological role: on the one hand, it modulates central appetite control mechanisms. Its anorexigenic effect is achieved by binding to GLP-1 receptors in the brain and slowing gastric emptying, which promotes feelings of satiety. On the other hand, it influences glucose homeostasis, lowering glucose concentrations in both fasting and postprandial periods, mainly by stimulating glucose-dependent insulin secretion and inhibiting glucagon secretion. GLP-1 agonists mimic the action of GLP-1 by binding to the GLP-1 receptor, thereby activating it much like the natural hormone [279–281].

The efficacy of these drugs in clinical trials often exceeds their efficacy in the real world. The discrepancy is due to a number of reasons, including non-adherence (up to 70.1% of patients discontinue therapy after 24 months) and an unrepresentative selection of patient groups [282]. In contrast, a 2025 meta-analysis found that, despite their efficacy in reducing body weight in those taking certain GLP-1 agonists, much of the weight loss was attributable to a reduction in lean body mass, which may be a disadvantage [283].

One randomised controlled trial [284] demonstrated that GLP-1 levels were higher in people on the ketogenic diet than in control groups. At the same time, the KD is known to be effective in suppressing hunger, enhancing satiety, reducing blood glucose levels, and improving glycaemia management [21]. It is therefore not surprising that both the ketogenic diet and GLP-1 agonists show similar weight loss effects [285]. Both are also effective in reducing blood glucose levels, which implies that their effects can be synergistic. Therefore, in individuals simultaneously using the KD and GLP-1 analogues, the drug dosage can probably be reduced without compromising the therapeutic efficacy. In fact, it is known that GLP-1 analogues may be associated with hypoglycaemia [286], which should be kept in mind when combining with the KD. Low-carbohydrate diets have been shown to help patients maintain glycaemic and weight control even after discontinuing these drugs [287,288].

### 7.4. Insulin and sulphonylurea derivatives

Both insulin and sulphonylurea derivatives are diabetes drugs used primarily to lower serum glucose concentrations. At the same time, it is known that KD lowers blood glucose concentrations so effectively that it can lead to hypoglycaemia when combined with these drugs. Therefore, their dosage may need to be adjusted (reduced), or discontinued altogether in KD users [177,179–183]. This issue is also addressed in section 4.1, as the use of hypoglycaemic drugs in combination with KD is one of the situations that requires special caution. This is because under conditions of reduced carbohydrate intake, the efficacy of these drugs may potentially be too strong, which may increase the risk of hypoglycaemia and hence requires careful monitoring and possible dose modification.

### 7.5. Antiepileptic drugs

Antiepileptic drugs are commonly used in treating epilepsy, and some are also often used in psychiatry for the treatment of mental illnesses such as bipolar disorder. These drugs can be divided into those that induce hepatic enzymes (e.g. phenytoin, phenobarbital and carbamazepine) and those that do not (e.g. topiramate, zonisamide, sodium valproate, lamotrigine, pregabalin or levetiracetam) [197]. These drugs do not always produce the clinically expected results, for example, in cases of drug-resistant epilepsy, where a ketogenic diet may be more effective. For this reason, the KD and antiepileptic drugs are often used concomitantly, raising the legitimate question of potential drug-KD interactions. Possible interactions of KD with selected antiepileptic drugs are listed below. Please note that other potential interactions are certainly possible.

Topiramate is mainly used as monotherapy and adjunctive therapy in the treatment of epilepsy, as well as in psychiatric disorders and in the treatment of migraine disorders [289]. Zonisamide is mainly used as adjunctive therapy in epilepsy in patients over 16 years of age [290]. Both drugs inhibit carbonic anhydrase, which can lead to metabolic acidosis due to reduced serum bicarbonate levels. Indications are that the risk of metabolic acidosis may be higher in the early phase of adaptation to the ketogenic diet. This was demonstrated in one study in which the KD was introduced in patients who were already on topiramate therapy. Serum bicarbonate levels decreased, albeit mainly in the adaptation period. The authors concluded that the combination therapy of topiramate with the ketogenic diet requires careful monitoring of bicarbonate levels. If any negative symptoms are observed, bicarbonates should be supplemented [291]. This issue was also addressed in a more recent paper pointing out not only the risk of metabolic acidosis developing as a result of the interaction of KD with topiramate or zonisamide, but also suggesting that the combination of KD with zonisamide (but not topiramate) may increase the risk of nephrolithiasis [292]. However, the authors emphasise that there is no need to avoid the concomitant use of KD with these drugs. It is possible that, due to their overlapping mechanisms of action, the combination of the ketogenic diet and zonisamide may have a potential additive effect, potentially enhancing therapeutic efficacy without the need to intensify pharmacological treatment.

In addition, KD may increase the activity of cytochrome P450 enzymes and thus potentially interact with anti-epileptic drugs metabolised by these enzymes. One of the results will be a reduction in the blood concentrations of these drugs. The literature reports reductions in the concentrations of lamotrigine, topiramate and lacosamide, as well as significant reductions in the concentrations of carbamazepine, valproic acid, and clobazam (which was also shown to be reduced by 42% in another study [293], but no change in the concentrations of oxcarbazepine, zonisamide, and levetiracetam after 4–12 weeks of MAD [294].

Valproic acid is itself a fatty acid that can be burned by cells as an energy source, and this process may be enhanced during KD, when fat metabolism accelerates. This may be one reason why valproate blood levels tend to decrease on a KD. Monitoring of serum valproate levels is recommended as dosage adjustments may be clinically indicated when valproate and the KD are used together [295,296].

Lithium is a naturally occurring mineral with mood-stabilizing properties that is often used in the management of bipolar disorder. As discussed earlier, the ketogenic diet changes the balance of fluid and electrolytes in the body, and this is largely due to increased renal excretion of sodium and water. Lithium is filtered and excreted by the kidneys, which handle lithium in a similar way to sodium [297,298]. The combination of lithium and the KD may therefore result in changes to blood lithium levels, particularly during the initial keto-adaptation period. Since lithium has a narrow therapeutic window,monitoring of lithium levels before and during KD therapy is recommended.

### 7.6. Diuretics

Diuretics are drugs that increase diuresis (urine excretion). They are used, for example, in treating oedema, heart failure, hypertension, and ascites. They increase urine production and volume and thus intensify excretion of water and electrolytes (with some exceptions) from the body [299]. Given that the period of adaptation to the ketogenic diet produces similar effects (increased diuresis and loss of electrolytes) [218], initiating the KD in patients taking diuretic drugs may potentiate these effects, thereby potentially disrupting water and electrolyte balance. The pharmacodynamics of thiazide diuretics may also make it more difficult to achieve the state of ketosis, by increasing blood glucose levels [197].

### 7.7. Lipophilic drugs

Lipophilic drugs are fat-soluble compounds with low water solubility that can penetrate biological barriers (e.g. the blood-brain barrier) [300]. This class of drugs includes a broad spectrum of different types of drugs [197]: olanzapine and clozapine (classified as antipsychotics) [301]; amitriptyline, nortriptyline and doxepin (classified as antidepressants) [302–304]; diazepam and midazolam (categorised as benzodiazepines) [305]; zolpidem and zopiclone (categorised as sedatives) [306,307]; phenytoin, carbamazepine, valproic acid, gabapentin and pregabalin (categorised as antiepileptics) [308,309]; simvastatin, fluvastatin, lovastatin, pitavastatin, and atorvastatin (classified as hypolipemic drugs) [310]; propranolol and metoprolol (classified as beta-blockers) [311]; amiodarone (classified as an antiarrhythmic) [312]; and a number of others, such as chloroquine and mefloquine (antimalarial drugs); ketoconazole and itraconazole (antifungal drugs); spironolactone (a diuretic); rifampicin (an antituberculosis drug); ivermectin (an antiparasitic drug); cetirizine and loratadine (antihistamines); methadone (an opioid); ritonavir and saquinavir (antivirals); and tacrolimus (an immunosuppressant) [197].

Highly lipophilic drugs may show increased bioavailability when administered in combination with high-fat diets [313]. Therefore, the KD may increase their absorption, which could increase their serum concentrations; this could increase their therapeutic efficacy but also increases the risk of adverse effects. In such cases, the physician must use their clinical judgment to make adjustments in drug dosages as needed. with the aim of achieving maximal efficacy with minimal adverse effects [197].

### 7.8. Corticosteroids

Corticosteroids are synthetic analogues of the natural steroid hormones produced by the adrenal cortex and have applications in a wide range of medical situations. This class of drugs is divided into glucocorticoids (which have immunosuppressive, anti-inflammatory and vasoconstrictive effects) and mineralocorticoids (which control the body’s water and electrolyte balance) [314].

These drugs may interact with the KD, as common side effects of corticosteroids are increased blood glucose concentrations (due to increased hepatic gluconeogenesis), decreased insulin sensitivity, and decreased glucose utilisation by adipose and muscle tissue [315]. In this case as well, elevated insulin levels play a role, as insulin is anti-ketogenic. High blood glucose is one of the main factors hindering the optimal state of nutritional ketosis. Namely, it is known that the higher the glucose to ketone ratio, the higher the glucose ketone index (GKI), which in turn adversely affects the depth of ketosis [316]. Moreover, the KD can increase cortisol levels [Whittaker et al. 2022] and activate the hypothalamic-pituitary-adrenal (HPA) axis [317], particularly in the short term, while systemic corticosteroids can lead to hypercortisolemia and inhibition of the HPA axis, another potential interaction of these drugs with KD [197,318]. Additionally, some findings point out to a possibly higher risk of water-electrolyte imbalance [197].

## 8. Possible side effects of KD

Potential side effects of the ketogenic diet are most often related to the process of the body adapting to the state of ketosis in the initial period of the diet. According to the literature, these side effects are typically observed in the first 2–3 days of starting the KD, most or all of them resolve within 2 to 4 weeks, and they are rarely severe enough to require discontinuation of KD [218]. In a retrospective study of 226 people who had followed a KD, reported side effects included dizziness (mild, 39.8%; moderate, 27.4%; severe, 11.5%), polyuria (72.1% overall), lethargy (69.7%), and nausea (mild, 29.2%; moderate, 16.4%; severe, 5.8%). The authors remarked that 90.3% of these subjects were satisfied with the KD, and 81.9% would recommend this dietary approach to anyone wishing to reduce body weight. Thus, despite some transient side effects, the overall perception of KD was very positive [319]. Another study evaluated personal experiences with KD shared by 300 online forum users. The most common side effects (in order of descending frequency) were: flu-like symptoms (44.6%), headache (24.8%), fatigue (17.8%), nausea (15.8%), dizziness (14.9%), brain fog (10.9%), gastrointestinal discomfort (10.9%), decreased energy (9.9%), feeling faint (7.9%), heartbeat alterations (5.9%), sore throat (5.9%), decreased appetite (5.0%), shaking (5.0%), body aches (4.0%), cravings (4.0%), cramping (3.0%), hunger (3.0%), increased thirst (3.0%), insomnia (3.0%), irritability (3.0%), muscular soreness (3.0%), muscular weakness (3.0%), panic (3.0%), sluggishness (3.0%), anxiety (2.0%), bloating (2.0%), dehydration (2.0%), diarrhoea (2.0%), dry mouth (2.0%), fever (2.0%), joint pain (2.0%), lethargy (2.0%), listlessness (2.0%), weak fingernails (2.0%), acne (1.0%), asthma (1.0%), chills (1.0%), constipation (1.0%), coughing (1.0%), decreased motivation (1.0%) and a few others, such as migraine, ears blocked, muscle tightness, vomiting, and phlegm in throat. Side effect reports peaked in the first week and decreased after four weeks [320]. However, this was a study based on an analysis of subjective opinions published on online forums, which limits its scientific value. More authoritative data was presented by Cervenka et al. who analysed a survey among an international panel of experts with clinical experience in the use of the ketogenic diet in adults, which allowed for a less biased and more standardised assessment of the diet’s effects. However, the results of this study cannot be extrapolated to the general population, as it was mainly concerned with KD side effects in patients with neurological disorders. For example, weight loss in that context was listed as a side effect, while it is the main goal (and therefore viewed as a benefit) of the KD in the general population. The study by Cervenka et al. estimated that the most common side effects of KD included (in descending order) constipation, weight loss, hyperlipidaemia, leg cramps, irregular menstruation (in women of childbearing age), nausea, and vomiting [321]. A systematic review published in 2025 analysed the available scientific literature on the prevalence of symptoms, as well as possible mechanisms and proposed interventions to alleviate these symptoms of the keto adaptation period. Children and adult populations were analysed separately. The most common symptoms in adults and children were constipation (1–68% in adults and 15–63% in children), nausea (8–16% and 27–42%), vomiting (1% and 5–36%), diarrhoea (2–23% and 3.6–20%), feelings of decreased energy (18–25% and 17–28%) and mood changes (1% and 6.7–10%), respectively. Symptoms identified in adults only were halitosis (38%), liver function abnormalities (24%), light-headedness/dizziness (15–21%), headache (8–25%), brain fog/reduced cognitive performance (10%) and skin rash (13–21%). Conversely, the side effects observed in children only included hypoglycaemia (6–28%), ketoacidosis (2–4%) and kidney stones (2.5–4%). It is known, however, that adequate preparation for the KD helps avoid a large proportion of symptoms, and that most of these symptoms resolve within 2–4 weeks anyway [218]. It is also worth noting from clinical practice that it is extremely rare for a patient on a ketogenic diet to experience dramatic weight loss accompanied by a rising HbA1c level - an atypical scenario, as HbA1c would typically be expected to decrease. Such a pattern may suggest underlying insulin deficiency or, in some cases, pancreatic cancer.

**Figure 2.**
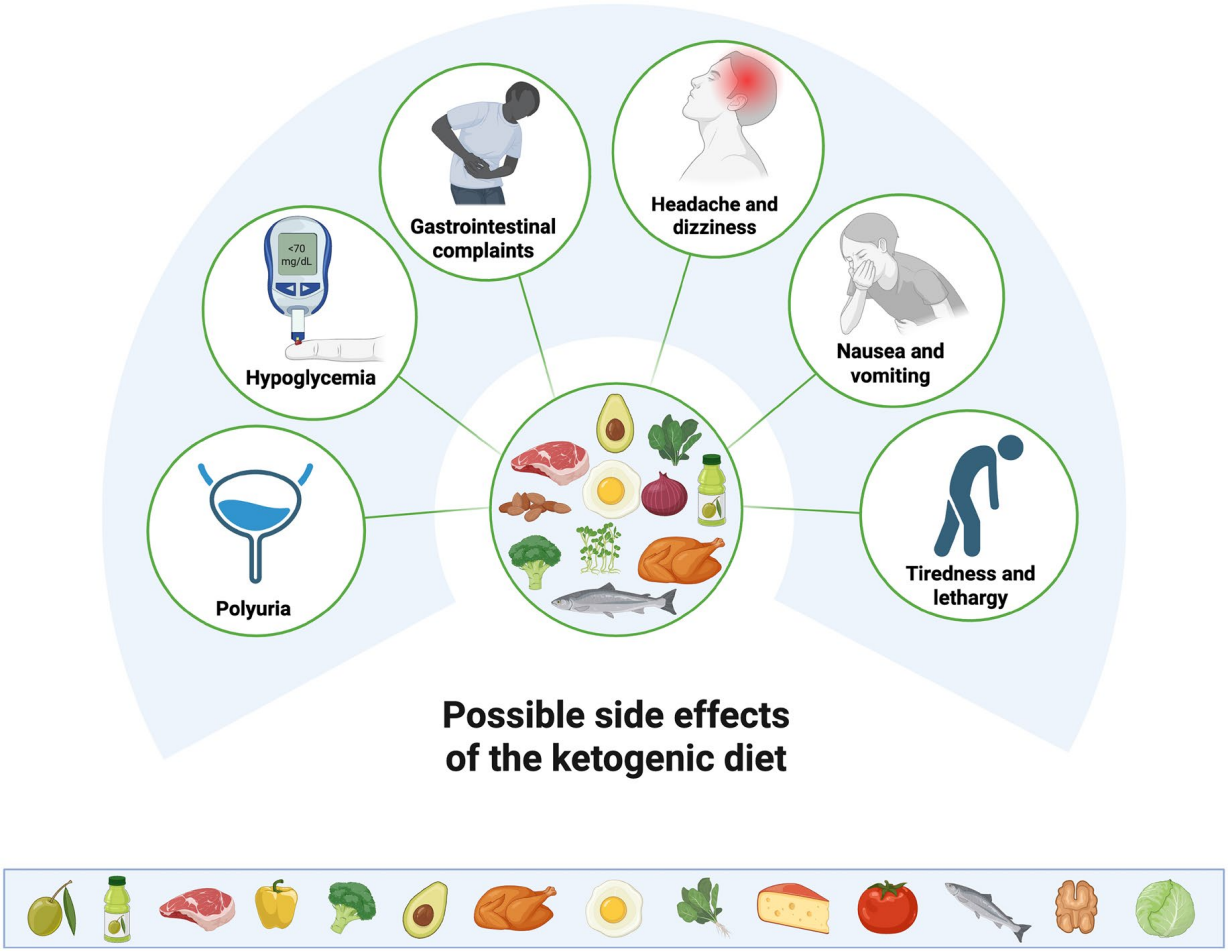
Possible side effects of the ketogenic diet. Created in BioRender https://BioRender.com/4rkpsp0.

Figure 2 The potential adverse effects of the ketogenic diet are presented in detail in Table 2, while the most common of them are illustrated in a more general, visual manner in Figure 2.

**Table 2.** Possible side effect of ketogenic diet.

Possible side effect of ketogenic diet			
Populations	Comments	Frequency of all reported side effects	Sources
Adults and children	A scoping review based on 89 studies	Both adults and children: constipation (1–68% in adults / 15–63% in children), nausea (8–16% / 27–42%), vomiting (1% / 5–36%), diarrhoea (2–23% / 3.6–20%), decreased energy/fatigue (18–25% / 17–28%) and mood changes (1% / 6.7–10%). Adults only: halitosis (38%), abnormal liver function tests (24%), dizziness/light-headedness (15–21%), headache (8–25%), brain fog/reduced cognitive performance (10%) and skin rash (13–21%). Children only: hypoglycaemia (6–28%), ketoacidosis (2–4%) and kidney stones (2.5–4%).	[218]
Adults	Retrospective observational study involving 226 individuals (*n* = 226)	Dizziness (mild, 39.8%; moderate, 27.4%; severe, 11.5%), polyuria (72.1% overall), lethargy (69.7%), and nausea (mild, 29.2%; moderate, 16.4%; severe, 5.8%)	[319]
Adults	Qualitative content analysis of online forums (43 forums, 300 unique users (*n* = 300))	Flu-like symptoms (44.6%), headache (24.8%), fatigue (17.8%), nausea (15.8%), dizziness (14.9%), brain fog (10.9%), gastrointestinal discomfort (10.9%), decreased energy (9.9%), feeling faint (7.9%), heartbeat alterations (5.9%), sore throat (5.9%), decreased appetite (5.0%), shaking (5.0%), body aches (4.0%), cravings (4.0%), cramping (3.0%), hunger (3.0%), increased thirst (3.0%), insomnia (3.0%), irritability (3.0%), muscular soreness (3.0%), muscular weakness (3.0%), panic (3.0%), sluggishness (3.0%), anxiety (2.0%), bloating (2.0%), dehydration (2.0%), diarrhoea (2.0%), dry mouth (2.0%), fever (2.0%), joint pain (2.0%), lethargy (2.0%), listlessness (2.0%), weak fingernails (2.0%), and other symptoms reported by single users (1.0% each): acne, asthma, depressed mood, derealization, ears blocked, mental confusion, migraine, muscle tightness, phlegm in throat, photophobia, restlessness, run down, runny nose, sinus pressure, tingling in head, vomiting.	[320]
Adults with epilepsy or other neurologic disorders	International multicenter expert survey (20 centers treating >2000 adults)	Side effects (in descending order of frequency): constipation, weight loss, hyperlipidaemia, leg cramps, irregular menstruation (women of childbearing age), nausea, vomiting (quantitative data limited; only hyperlipidaemia reported in ∼1/3 of adults)	[321]

## 9. Potential problems with the KD in clinical practice

Obviously, to achieve the therapeutic effects of KD, patients must adhere to its principles, which can be challenging. Possible practical problems initiating and maintaining the ketogenic diet may include psychological, emotional and sociological factors. For example, the prospect of imagining a dinner plate with a minimum amount of carbohydrates can seem overwhelming to some; others struggle with quitting ultra-processed foods (especially if their relationship with those foods is addictive. Also, the fear of failure or standing out can lead to anxiety. In turn, some patients with bipolar disorder may experience temporary exacerbation of mood symptoms that precede sustained improvement [322]. In a social context, adherence to the KD often involves the need to forgo traditional meals at family gatherings, which can be a significant challenge, especially in cultures where such meals play an important social role. In addition, people who prefer to eat out may have problems finding restaurants that offer suitable meals on their menus. For adolescents and adults who seek to conform to the dietary norms prevailing in their peer groups, a sense of exclusion may arise. People on the KD may find that avoiding carbohydrates leads to tense and stressful interactions with family members and friends, which can manifest as intrusive questions, disapproval, and even warnings from those who are less knowledgeable about the KD. The root cause of such reactions is that the principles of the KD conflict with the common belief that balanced diets are healthiest [322–324].

## 10. Strengths and limitations

This narrative review provides a comprehensive overview of the available literature, including meta-analyses, systematic reviews, randomized controlled trials, clinical studies, case reports, mechanistic evidence, reference databases (e.g. Orphanet), and clinical guidelines. The search strategy allowed identification of evidence on specific contraindications and safety concerns that may not be captured by broader keyword searches. To our knowledge, no other publication has addressed this topic so broadly from multiple perspectives, making this review a uniquely comprehensive contribution to the field.

As this is a narrative review, the selection of evidence does not follow a predefined systematic protocol; therefore, the review may be susceptible to selection bias. Additionally, the heterogeneity of included studies and reliance on selected databases, guidelines, and keywords may limit the generalizability of the findings, and evidence on rare conditions or specific drug interactions remains scarce.

## 11. Conclusions

The ketogenic diet is a promising tool for both prevention and treatment of not only certain neurological disorders, but also a number of metabolic disorders and diseases such as obesity, overweight, type 2 diabetes, and MAFLD. It has also gained recognition in the growing field of metabolic psychiatry. However, in certain disorders, diseases, or clinical situations, the ketogenic diet is contraindicated, whether absolutely or relatively. Furthermore, in some settings, the application of this dietary approach must be accompanied by special precautions.

Most absolute contraindications to the KD are specific rare genetic predispositions, mainly involving difficulties in converting fats into energy. These absolute contraindications include pyruvate carboxylase (PC) deficiency; disorders of fatty acid beta-oxidation (i.e. primary carnitine deficiency or PCD), carnitine palmitoyltransferase deficiency, carnitine-acylcarnitine translocase (CACT) deficiency, 3-hydroxyacyl-coenzyme A dehydrogenase (HADH) deficiency, medium-chain 3-hydroxyacyl-CoA dehydrogenase deficiency (MHADD), long-chain 3-hydroxyacyl-CoA dehydrogenase deficiency, medium-chain acyl-CoA dehydrogenase deficiency (MCADD), very-long-chain acyl-CoA dehydrogenase deficiency (VLCADD); and porphyria.

Relative contraindications are based on some literature findings and/or indirect mechanisms that make KD potentially risky. Relative contraindications include a number of situations, such as acute pancreatitis (AP), acute liver failure (ALF), advanced stages of chronic kidney disease (CKD), use of propofol, and familial hypercholesterolaemia (FH). What makes these different from absolute contradictions is that the KD can potentially be a valid and potentially benefical option in these relatively contraindicated situations, but relevant research and clinical experience is often lacking. As a precaution, it may be safe to consider these situations absolute contraindications until they have been thoroughly researched and any outstanding safety questions resolved.

Situations requiring special caution, while not necessarily absolute contraindications to the KD, call for a tailored and carefully planned approach. This includes, but is not limited to, an in-depth knowledge of potential interactions and risks, close monitoring of the patient’s health status, increased clinical supervision, adjustment - and in some cases discontinuation - of medication doses, an appropriately designed nutrition plan, and possibly increasing the proportion of selected nutrients in the diet and/or targeted supplementation. It is likely that in some of these cases the KD could fail, but in others (e.g. in patients with type 2 diabetes taking hypoglycaemic drugs) it may offer a number of benefits, provided that medication doses are monitored and adjusted (or discontinued) by a specialist. Situations in which special care and attention should be exercised include (but are not limited to) the following: T2DM treated with hypoglycaemic medication (not metformin or GLP-1 drug), T1DM, hypertension treated with antihypertensive medication, cholelithiasis, gallbladder removal, electrolyte deficiency, cardiac arrhythmia, pregnancy, breastfeeding, underweight, intense physical activity, periods of intense stress, and postoperative recovery.

The ketogenic diet may interact with certain medications, which can lead to a range of both benefits and risks. Interacting substances include SGLT-2, metformin, GLP-1 agonists, insulin, sulphonylurea derivatives, antiepileptics, diuretics, lipophilic drugs, and corticosteroids.

Adverse effects associated with the KD are generally transient; they most often occur during the initial period of the body’s adaptation to the new metabolic state, and typically resolve within a few weeks. The most commonly reported side effects include the so-called ‘keto flu’, fatigue, nausea, lethargy, headaches, dizziness, gastrointestinal complaints (such as constipation or diarrhoea), lack of energy, polyuria,and hypoglycaemia. Importantly, these can often be prevented if switching to KD is preceded by adequate preparation.

This review seeks to serve as a useful reference for clinical practice by presenting a detailed consideration of clinical and metabolic safety concerns related to the KD, its possible side effects, and potential interactions between the KD and commonly prescribed medications. At the same time, it should be noted that the contraindications we mention do not exhaust the full spectrum of possible clinical issues that may arise with the ketogenic diet. Please also note that the assignment of contraindications to absolute or relative categories sometimes varies between literature sources, often based on authors’ subjective clinical interpretation and experience; therefore, clinicians are advised to use their clinical judgment when considering the use of the KD in these clinical scenarios and tailor their treatment plan to the specific needs of their patients.

## Data Availability

There is no data associated with this research
